# Epigallocatechin gallate (EGCG) inhibits lipopolysaccharide‐induced inflammation in RAW 264.7 macrophage cells via modulating nuclear factor kappa‐light‐chain enhancer of activated B cells (NF‐*κ*B) signaling pathway

**DOI:** 10.1002/fsn3.3427

**Published:** 2023-05-25

**Authors:** Imam Hossen, Zhang Kaiqi, Wu Hua, Xiao Junsong, Huang Mingquan, Cao Yanping

**Affiliations:** ^1^ Beijing Technology and Business University Beijing China; ^2^ Beijing Advanced Innovation Center for Food Nutrition and Human Health Beijing China; ^3^ Beijing Engineering and Technology Research Center of Food Additives Beijing China; ^4^ Key Laboratory of Brewing Molecular Engineering of China Light Industry Beijing China

**Keywords:** anti‐inflammation, EGCG, LPS, macrophages, NF‐κB

## Abstract

Epigallocatechin‐3‐gallate (EGCG) is a major bioactive compound in tea polyphenol extract. After ingestion, EGCG reaches the intestine and may commence anti‐inflammation in the intestinal organ. Thus, in this paper, the anti‐inflammatory effect of EGCG was studied using lipopolysaccharide (LPS)‐induced inflammation in RAW 264.7 cells. LPS induction instigated morphological deformation extensively which was normalized by EGCG. In LPS‐induced macrophage cells, EGCG was found to lower cellular nitric oxide (32% of LPS group) and intercellular ROS level (45.4% of LPS group). It also suppressed the expression of IL‐1β (LPS 132.6 ± 14.6, EGCG 10.67 ± 3.65), IL‐6 (LPS 2994.44 ± 178.5, EGCG 408.33 ± 52.34), TNF‐α (LPS 27.11 ± 2.84, EGCG 1.22 ± 0.03), and iNOS (LPS 40.45 ± 11.17, EGCG 10.24 ± 0.89). The GO function analysis identified that these differential genes involved 24 biological processes, 18 molecular functions, and 19 cellular component‐related processes. KEGG pathway enrichment analysis revealed that LPS significantly affects NF‐κB, TNF, and TLR signaling pathways. Western blotting revealed that EGCG diminished P‐IκB/IκB ratio by 75% and p‐p65/p65 by 50% compared to the LPS group. Finally, Arg‐1 and CD‐206 mRNA expression were determined by RT‐PCR, which was consistent with the RNA‐Seq result. These findings indicate that EGCG exerts an anti‐inflammatory effect by reducing NO and ROS production, suppressing TLR4 protein expression, and inhibiting IκB and p65 phosphorylation.

## Graphical abstract

Epigallocatechin‐3‐gallate (EGCG) is a major bioactive compound in tea polyphenol extract. In LPS‐induced macrophage cells, EGCG was found to lower cellular nitric oxide (32% of LPS group) and intercellular ROS level (45.4% of LPS group). It also suppressed the expression of IL‐1ß (LPS 132.6 ± 14.6, EGCG 10.67 ± 3.65), IL‐6 (LPS 2994.44 ± 178.5, EGCG 408.33 ± 52.34), TNF‐a (LPS 27.11 ± 2.84, EGCG 1.22 ± 0.03), and iNOS (LPS 40.45 ± 11.17, EGCG 10.24 ± 0.89). Western blotting revealed that EGCG diminished P‐IκB/IκB ratio by 75% and p‐p65/p65 by 50% compared to the LPS group. Finally, Arg‐1 and CD‐206 mRNA expression were determined by RT‐PCR, which was consistent with the RNA‐Seq result.

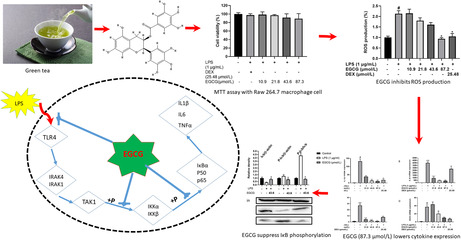

## 1 INTRODUCTION

Inflammation, which is the first line of defense of our body against pathogens, irritation, and injury, is a sophisticated response regulated by cytokines and chemokines. Various immune cells including monocytes, neutrophils, and macrophages are acquired by our innate immune system to address the inflammation (Novilla et al., 2017; Oyungerel et al., 2013). Macrophage cells play a vital role in inflammation modulation, as they are the first responders to inflammation. Exposure to cytokines, chemokines, or bacterial lipopolysaccharide (LPS) leads to macrophage cell activation. Phagocytic activity of macrophage cells increases many folds during inflammation and activated macrophage cells fight pathogens directly. Macrophage cells indirectly maneuver inflammation by secreting proinflammatory cytokines (IL‐1β, TNF‐α, IL‐6) and inflammatory mediators (NO, iNOS) (Arango Duque & Descoteaux, 2014). Unregulated secretion of cytokines and inflammatory mediators results in damage on both cellular and tissue levels. Cellular damage ends up in apoptosis and necrosis while tissue damage includes the development of many chronic diseases, viz., rheumatoid arthritis, chronic hepatitis, diabetes, pulmonary fibrosis, and cancer (Kim et al., 2016; Laveti et al., 2013; Liu et al., 2017; Tsai et al., 2018).

Nuclear factor kappa‐light‐chain‐enhancer of activated B cells (NF‐*κ*B) is one of the most important transcription factors that regulate inflammatory response along with other physiological processes (Hossen et al., 2020, 2021; Schulert & Grom, 2015). As long as an inhibitor of *κ*Bs (I*κ*Bs) is not phosphorylated, NF‐*κ*B remains inactive in the cytoplasm. Phosphorylation of I*κ*B leads to NF‐*κ*B nuclear translocation and ends up in transactivation of downstream genes (Baker et al., 2011; Siebenlist et al., 2005). Activated downstream target genes direct the cells to produce inflammatory cytokines and mediators including NO, TNF‐α, and IL‐6. They also direct the recruitment of innate immune cells to combat inflammation (Lawrence, 2009; Schneider et al., 2014). These make NF‐*κ*B an ideal candidate to study anti‐inflammatory chemicals and substances.

Green tea is very popular in East Asia mainly in China, Japan and gaining popularity around the world (Chacko et al., 2010). Previous studies have shown that the consumption of green tea may reduce risks associated with cardiovascular disease and exert numerous health benefits (Wang et al., 2011). Around 30% dry weight of green tea are polyphenols and among them, flavonoids are most important; 80%–90% of the flavonoids are catechins and epigallocatechin‐3‐gallate (EGCG), which cover 59% of the total catechins present (Jigisha et al., 2012; Reygaert, 2017; Roowi et al., 2010). EGCG has low bioavailability (0.1%) but its content in the intestine is very high. The bioavailability of EGCG is proportional to immune system upregulation and improvement of health. The more EGCG reaches the target site, the better it is for the body (Xu et al., 2015). Lambert et al. (2003) intragastrically fed male CF‐1 mice 163.8 μmol/kg EGCG and the levels in the small intestine and colon were 45.2 ± 13.5 and 7.86 ± 2.4 nmol/g, respectively. Accumulating studies have demonstrated that EGCG possesses numerous health benefits, including anti‐inflammatory, antioxidant, anticancer, and antitumor properties. EGCG lowers the pro‐inflammatory cytokine and chemokine expression; also suppresses MAPK, STAT, TLR4, and NF‐κB signaling pathway (Almatroodi et al., 2020; Cao et al., 2019; Chu et al., 2017; Yahfoufi et al., 2018).

Therefore, this study intends to check the effect of EGCG on suppressing body inflammation and the possible mechanisms involved. This is for the first time the effects of EGCG from morphological impact to the biochemical changes, gene expression level, and protein expression changes are combined in one single manuscript. We attempted to compare the effects of EGCG and dexamethasone (DEX) on inflammation in terms of cell phagocytic capacity and the inhibitory effects on IL‐1β, IL‐6, TNF‐α, and iNOS pro‐inflammatory factors effect. We also observed the cell morphology using an inverted microscope, measured physiological indicators such as NO and ROS, and used reverse transcription PCR (RT‐PCR) to determine the level of inflammatory factors to evaluate the anti‐inflammatory effect of EGCG. Additionally, transcriptome sequencing was used to determine the mRNA levels of related factors, and western blotting was used to determine related protein expression related to the possible anti‐inflammatory mechanism. Our findings will provide a more theoretical basis for the role of EGCG in maintaining intestinal permeability, and its later use in the development of healthy foods.

## 2 MATERIALS AND METHODS

### 2.1 Reagents

EGCG (HPLC grade, ≥98%) (Catalogue no. HY‐N6263) and 3‐(4,5‐Dimethylthiazol‐2‐yl)‐2,5‐diphenyltetrazolium bromide (MTT) (Catalogue no. BN30793) were purchased from Shanghai Yuanye Biotechnology Co., Ltd. Neutral red (Catalogue no. DE‐E895A) was purchased from Shanghai Macklin Biochemical Co., Ltd. Dulbecco's Modified Eagle Medium (DMEM) (Catalogue no. B20290), fetal bovine serum (FBS) (Catalogue no. P08X20), and glutamine (Catalogue no. BIO‐000001) were purchased from Gibco Corporation (Life Technologies, Thermo Fisher Scientific). Phosphate‐buffered saline (PBS) (Catalogue no. B20719) and trypsin (Catalogue no. 84278A) were obtained from HyClone. Dimethyl sulfoxide (DMSO) (Catalogue no. BN35879), 2′, 7’‐Dichlorofluorescin diacetate (DCFH‐DA) (Catalogue no. DE‐D1002), dexamethasone (Dex) (Catalogue no. E120263), and LPS (Catalogue no. S11060) were bought from Sigma‐Aldrich. Sodium pyruvate (Catalogue no. 113–24‐6) was obtained from Beijing Banxia Biological Technology Co., Ltd. Isopropanol (Catalogue no.67–63‐0), ethanol (Catalogue no. G00004), and glacial acetic acid (Catalogue no. A116166) were purchased from Beijing chemical works company Ltd. RNA extraction kit was purchased from TransGen Biotech Co., Ltd. SYBR Green PCR master mix and reverse transcription kit were purchased from Toyobo (Japan). Total nitric oxide (NO) assay kit was obtained from Beyotime Institute of Biotechnology Co., Ltd.

### 2.2 Cell culture

Raw 264.7 macrophage cells were collected from the Stem Cell Bank, Institute of Zoology (Chinese Academy of Sciences). Cells were maintained in DMEM supplemented with 10% FBS, 1% glutamine, and 1% sodium pyruvate. Cells were incubated at 37°C in a 5% CO_2_ atmosphere and sub‐cultured every 2 days (Hossen et al., 2021).

### 2.3 Cytotoxic and MTT assay

To prepare the EGCG stock solution, 1 mg of EGCG was dissolved in 1‐mL PBS and then stored at −20°C till use. Cell viability was measured by MTT assay following the method followed by Hossen et al. (2021). RAW 264.7 cells (1 × 10^5^ cells/mL) inoculated at 100 μL in 96‐well plates for 24 h. Later, the culture solution is replaced with a serum‐free medium mixed with different doses of EGCG and LPS (1 μg/mL) and Dex (25.48 μmol/L). After 24 h of incubation, the culture solution was discarded, and then MTT stock solution was added at 37°C and left for incubation for 4 h. The formazan crystals formed in this step were dissolved by adding 150‐μL DMSO and optical density (OD) is measured at 570 nm using a spectrophotometer (Tecan, Männedorf, Switzerland). The following formula is used to measure cell viability.

$$\text{cell viability}\;\left(\%\right)=\frac{{\mathrm{OD}}_{\text{Control}}-{\mathrm{OD}}_{\text{Sample}}}{O_{\text{Control}}-{\mathrm{OD}}_{\text{Blak}}}\times 10\%$$

### 2.4 Cell morphology analysis

For morphology analysis, 6.3 × 10^5^ RAW264.7 cells were seeded in a six‐well plate and incubated in a humidified incubator for 24 h at 37°C with 5% CO_2_ in it. Then, 43.6 μmol/L EGCG +1 μg/mL LPS, 25.48 μmol/L DEX + 1 μg/mL LPS, and 1 μg/mL LPS were added and incubated for 24 h. Later, an inverted microscope is used to randomly select three locations in the dish for morphological recording, and calculate the pseudo‐foot ratio (Hong et al., 2012).

### 2.5 Phagocytosis

RAW264.7 cells (1 × 10^5^ cells/mL) were seeded in a 96‐well plate and incubated for 24 h at 37°C in a 5% CO_2_ incubator until full confluency. After LPS treatment, the cells were added with EGCG (21.8 μmol/L, 43.6 μmol/L, and 87.2 μmol/L) and DEX (25.48 μmol/L), and incubated for 24 h. In the following, the cells were added with 100 μL of 0.1% neutral red solution and incubated for another 4 h. Later, the culture solution is discarded and washed thrice with PBS to remove neutral red that has not been engulfed by the cells. Then, 100 μL of acetic acid and ethanol solution (1:1, v/v) was added to each well, and the plates were placed at 4°C for 4 h to allow full lysis of the cells. Then, the absorbance of the cell lysate was measured using a spectrophotometer (Tecan) at 540 nm, and relative phagocytic activity was calculated by following the method of Chen et al. (2016).

### 2.6 Determination of NO content in cells

Griess method was applied to measure the NO content by following the method of Joo et al. (2014). Raw 264.7 cells (6.3 × 10^5^) grew to confluency in a 6‐cm cell culture dish containing a 3‐mL culture medium. After confluency, various concentrations of EGCG (10.9 μmol/L, 21.8 μmol/L, 43.6 μmol /L, and 87.2 μmol/L) and DEX (25.48 μmol/L) were added to cells pretreated with LPS (1 μg/mL). After 24 h of incubation, cell supernatants were separated by centrifugation at 1500x g, then 100 μL of supernatants from each type was added to 100‐μL Griess reagent. Absorbance was measured at 540 nm using a spectrophotometer (Tecan, Männedorf, Switzerland) in a 96‐well microplate reader, and NO concentration was calculated using the standard curve.

### 2.7 ROS level determination

DCFH‐DA fluorescent probe method was used to detect the ROS level (Hossen et al., 2021). RAW264.7 cells (1 × 10^5^ cells) were cultured in a 96‐well dark plate until confluency. Then, the culture medium was discarded and cells were washed gently with PBS buffer. Afterward, cells were treated with LPS (1 μg/mL) and then each group was treated with EGCG (10.9 μmol/L, 21.8 μmol/L, 43.6 μmol /L, and 87.2 μmol/L) and DEX (25.48 μmol/L). Then, cells were incubated for 24 h. DCFH‐DA was added with a final concentration of 10 μmol/L and incubated at 37°C in the dark for 30 min. Then, the cell medium was discarded and unbound DCFH‐DA was washed with PBS buffer. Then, 100‐μL cell medium was added and fluorescence intensity was measured with an excitation wavelength of 485 nm and an emission wavelength of 530 nm using a spectrophotometer (Tecan, Männedorf, Switzerland).

### 2.8 .2.8 Transcriptome analysis

RAW264.7 cells (6.3 × 10^5^ cells/mL) were cultured till adherence in a 6‐cm‐diameter cell culture dish. After the cells adhered to the wall, the serum‐free medium was used to replace the culture medium and then inoculated with LPS and EGCG + LPS for 24 h. After that, cells were washed twice with PBS buffer and 1‐mL Transzol (TransGen Biotech, Beijing) lysate was added to lyse the cells. After lysis, lysates were transferred to a 1.5 mL of RNase‐free centrifuge tube and stored at −80°C. Subsequent RNA extraction, detection, library construction, sequencing (Illumina HiSeq platform), and preliminary analysis were performed in Biomarker Technologies Corporation, Beijing. Sequenced data were filtered to get clean data after primary analysis, and compared the sequence with the mouse reference genome to get mapped data. Subsequent evaluation of library quality included insert length test, randomness test, and data saturation test as well as the analysis of sequence–structure levels such as variable splicing analysis, new gene discovery and gene structure optimization, and finally differential expression quantification of sample genes and difference analysis (Yu et al., 2018).

The expression level of the sample genes was calculated, and the FPKM algorithm was used to normalize the expression level:

$$\text{FPKM}=\frac{\text{cDNA fragments}}{\text{Mapped fragments}\;\left(\text{Millions}\right)\times \text{Transcript length}\;\left(\mathrm{kb}\right)}$$

cDNA fragments indicate the number of fragments aligned to the transcript; mapped fragments (millions) indicate the total number of fragments aligned to the transcript, in millions; Transcript length (kb) is the length of the transcript.

DEseq software was used for differential gene screening, and the screening criteria were fold change ≥2.0 (Log2 fold change ≥l) and FDR ≤0.01. Among them, fold change represents the ratio of expression between two groups of samples, *q*‐value is the significance of the differential expression, and the *p*‐value is corrected to obtain the FDR value of the false discovery rate. Controlling FDR below a certain threshold can reduce the false‐positive rate differential expression of genes.

### 2.9 Determination of IL‐1β, IL‐6, TNF‐α, and iNOS using RT‐PCR analysis

RT‐PCR analysis was conducted using a Bio‐Rad CFX96 touch system following the method described by Gao et al. (2020). The total RNA from the RAW 264.7 macrophages treated with LPS in the presence or absence of EGCG and DEX was extracted using the TransZol Up reagent (TransGen Biotech). Complementary DNA (cDNA) was synthesized from 1 μg of total RNA using a transcription kit (TOYOBO). NCBI blast and Primer 5 were used to design specific primer sequences for RT‐PCR. Primer sequences used for RT‐PCR are given in Table 1. The reaction conditions used for 38 cycles are as follows: predenaturation at 95°C for 2 min, denaturation at 95°C for 30 s, annealing at 57°C for the 30s, extension at 72°C for 30s, extension at 72°C for 2 min.

**TABLE 1:** Primer sequences used for RT‐PCR.

Name	Sequence
β‐Actin‐F	CCTAGAAGCATTTGCGGTGCACGATG
β‐Actin‐R	TCATGAAGTGTGACGTTGACATCCGT
IL‐1β‐F	TGCAGAGTTCCCCAACTGGTACATC
IL‐1β‐R	GTGCTGCCTAATGTCCCCTTGAATC
IL‐6‐F	AAGTGCATCATCATCGTTGTTCATACA
IL‐6‐R	GAGGATACCACTCCCAACAGACC
TNF‐α‐F	TACAGGCTTGTCACTCGAATT
TNF‐α‐R	ATGAGCACAGAAAGCATGATC
iNOS‐F	GCTGTGTGTCACAGAAGTCTCGAACTC
iNOS‐R	AATGGCAAACATCAGGTCGGCCATCATC
Arg‐1‐F	CAAGACAGGGCTCCTTTCAG
Arg‐1‐R	GTAGTCAGTCCCTGGCTTATGG
CD206‐F	CCTCAACCCAAGGGCTCTTCTAA
CD206‐R	AAGGTGGCCTCTTGAGGTATGTG

### 2.10 Quantification of 67LR, IκB, p65, PPARγ, and TLR4 using western blotting

Western blot analysis was conducted for protein expression as described by Hossen et al. (2020). RAW264.7 cells (8.5 × 10^5^ cells/mL) were inoculated in a 6‐cm cell culture dish and incubated for 24 h. Later, LPS or LPS + EGCG was added for 24 h. Then, cells were washed twice with PBS and protein was extracted using cell lysate. The protein concentrations were determined by the BCA method. An equal amount of protein (50 μg) was separated by 10% SDS‐PAGE at 80 V for 120 min. Separated proteins are then transferred to a PVDF membrane (Millipore), after blocking the membrane with 5% nonfat milk prepared in Tris‐buffer saline mixed with 0.1% Tween 20 (TBST) for 2 h at room temperature. Primary and secondary antibodies for β‐actin, IκB, p IκB, p65, p‐p65, and PPARγ were obtained from Cell Signaling Technology. TLR4 and 67LR were purchased from Santa Cruz Biotechnology, Inc. Primary antibodies were used at 1:1000 dilution and secondary antibodies at 1:2000 dilution. The membrane was incubated with the primary antibodies at 4°C overnight. Later, the membrane was washed twice with TBST and incubated with the secondary antibodies at 37°C for 1 h. The protein bands were visualized using the ECL detection kit (Beijing BioDee Biotechnology Company Ltd) with a chemiluminescence system.

### 2.11 Data processing

Screening and mapping of differential genes of transcriptome sequencing data were completed using various data processing tools provided by the Bimaike cloud platform. Excel 2010 was used to process the data, and then SPSS 17.0 was used for one‐way analysis of variance. Finally, GraphPad 7 was used for plotting. Fisher's LSD test was used to corroborate the differences that occur among groups.

## 3 RESULTS AND ANALYSIS

### 3.1 Cytotoxicity and MTT assay

**FIGURE 1:**
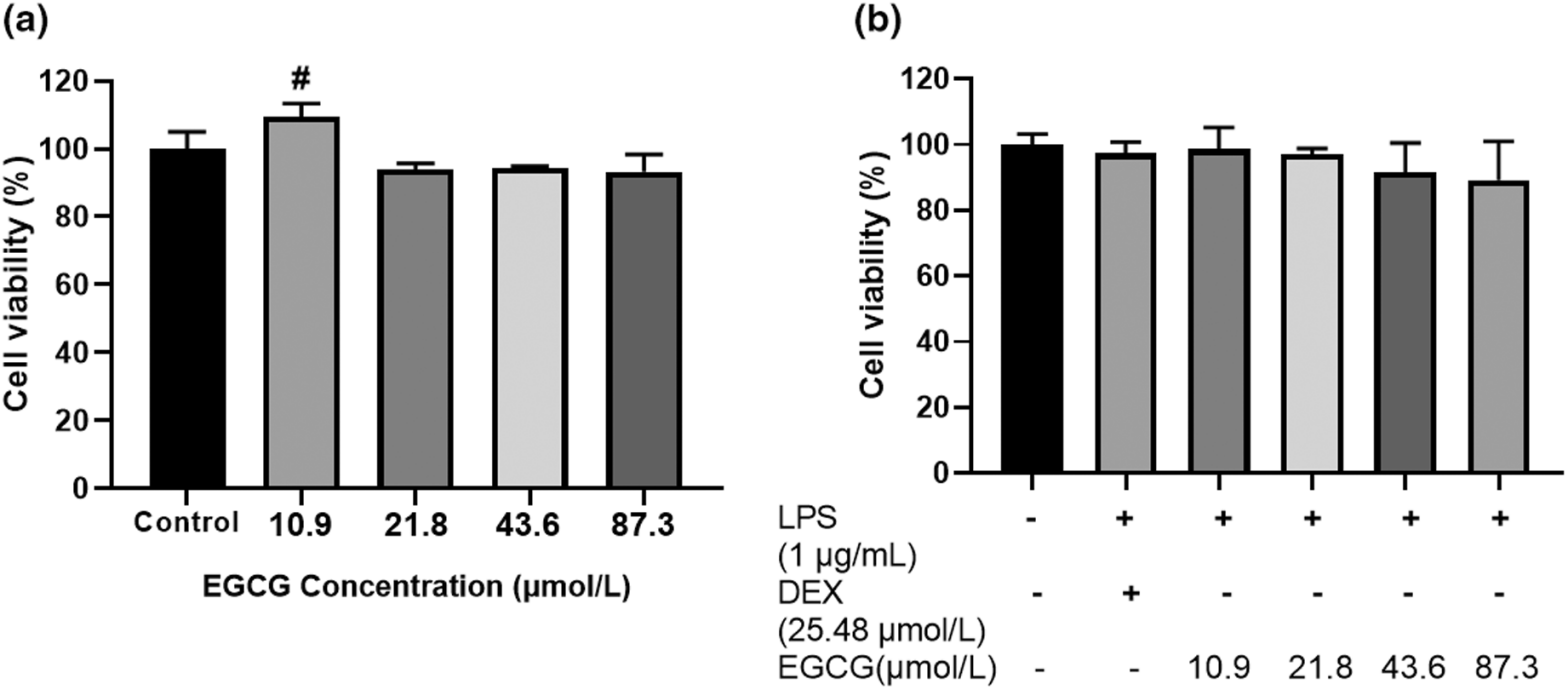
RAW264.7 macrophage cells (1 × 10^5^ cells) cultured and treated with EGCG and EGCG+LPS and DEX for 24 h. Effect of EGCG and LPS on the survival rate of RAW264.7 macrophage cells (^#^ *p* < .05 vs. control). Each data point represents the mean ± SD (*n* = 3).

Before experimenting with EGCG, we attempted to evaluate the cytotoxic effects of EGCG with different doses. RAW264.7 cell survival rate was determined by MTT assay. After 24 h of incubation of cells with EGCG (Figure 1a), DEX + LPS, and EGCG + LPS (Figure 1b) at 90% and above, it showed no obvious toxic effect on cells. After treating cells with 10.9 μmol/L EGCG for 24 h, the cell survival rate reached about 109%, which was significantly higher than that of the control group (*p* < .05). It is evident that the concentrations of EGCG (10.9–87.3 μmol/L) used in the subsequent experiments are within the safe range and have no effect on cell survival.

### 3.2 Cell morphology of raw 264.7

**FIGURE 2:**
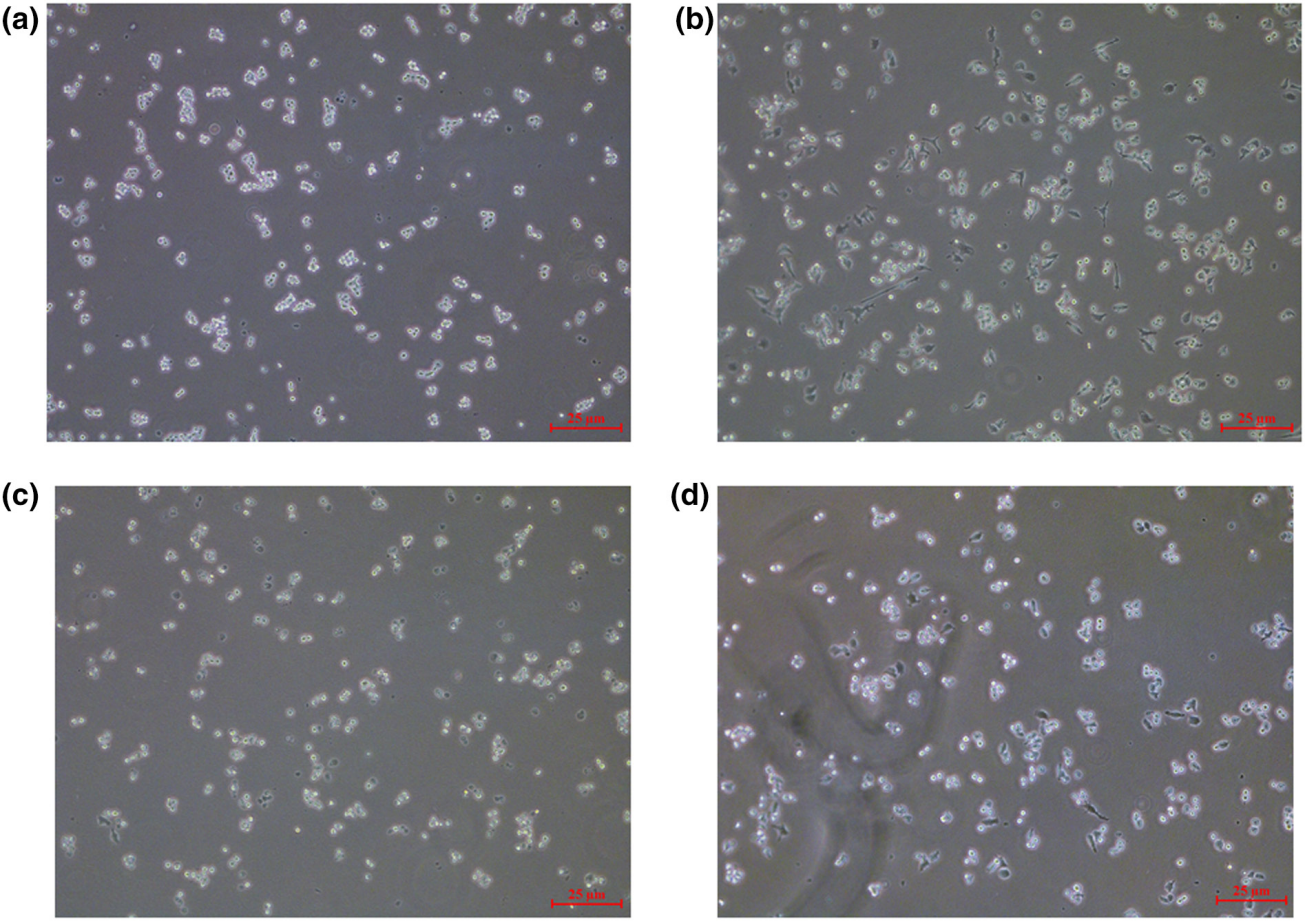
RAW264.7 macrophage cells (1 × 10^5^ cells) cultured and treated with EGCG and EGCG+LPS and DEX for 24 h. Morphological changes in RAW264. 7 cells were observed in an inverted microscope. (a). control, (b). LPS, (c). EGCG, (d). DEX.

Figure 2 represents the cell morphology of the macrophage RAW 264.7 cells under EGCG and DEX treatment in the presence or absence of LPS (1 μg/mL). Cells were then observed and photographed with an inverted microscope after 24 h of incubation and then the pseudo‐foot ratio was calculated. The cells in the control group were mostly round and bright (Figure 2a), and the LPS‐treated cells formed long and slender pseudopods (Figure 2b), which were different from the normal cells. After EGCG (Figure 2c) and DEX (Figure 2d) treatment, most of the cells were still round, significantly inhibiting LPS‐induced morphological changes.

**FIGURE 3:**
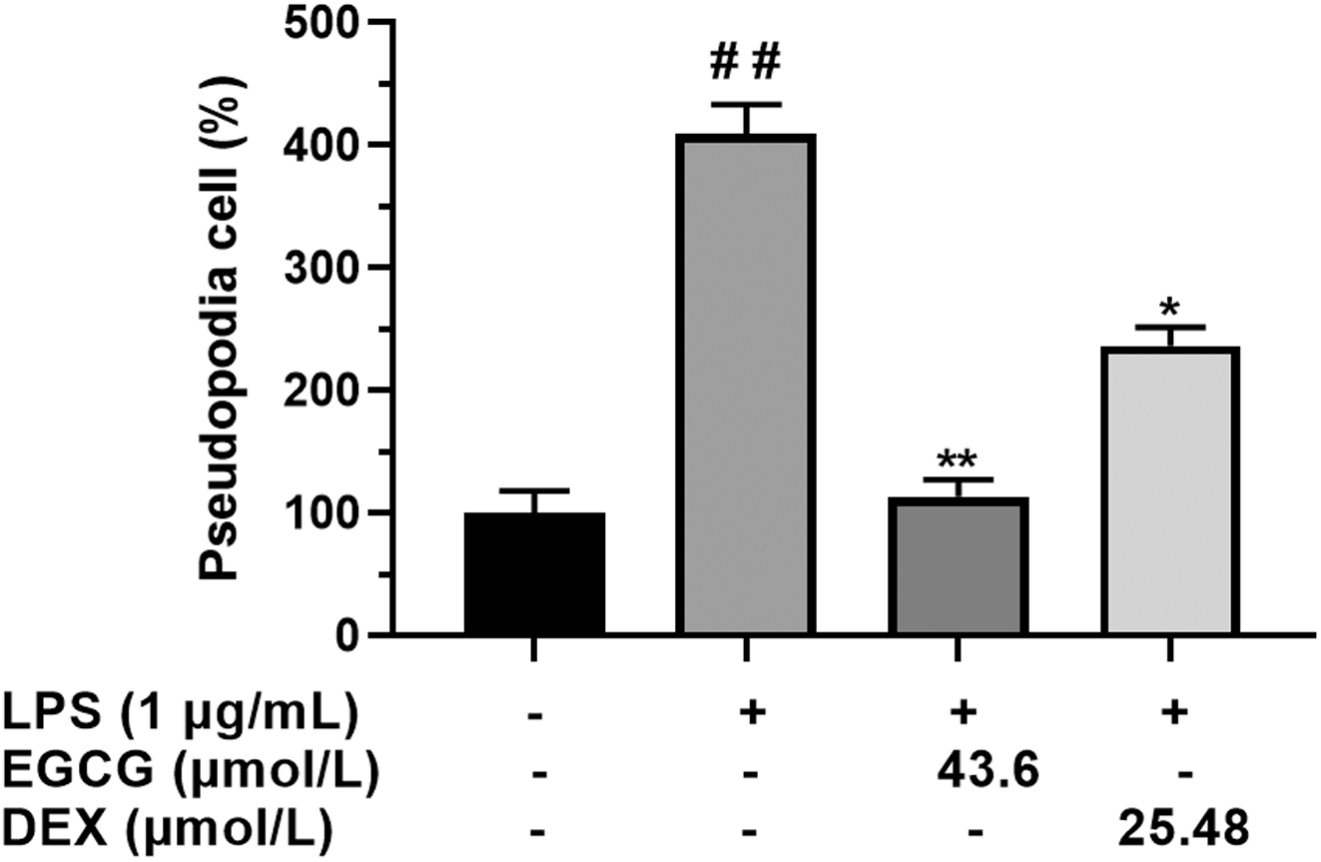
RAW264.7 macrophage cells (1 × 10^5^ cells) cultured and treated with EGCG and EGCG+LPS and DEX for 24 h. Percentage of cells with pseudopods was calculated using an inverted microscope. ^##^ *p* < .01 versus control group; *p < .05 versus LPS group, **p < .01 versus LPS group. Each data point represents the mean ± SD (*n* = 3).

The number of pseudopods in the LPS group was about four times higher than that of the control group (*p* < .01). EGCG significantly inhibited the generation of pseudopods caused by LPS, the number of pseudopods in the EGCG group was about 110% (*p* < .01). The number of pseudopods in the DEX group was about 220%, which was significantly lower than that in the LPS group, but it was still about twice that of EGCG (*p* < .05) (Figure 3).

### 3.3 Effects of EGCG on LPS‐induced phagocytosis

**FIGURE 4:**
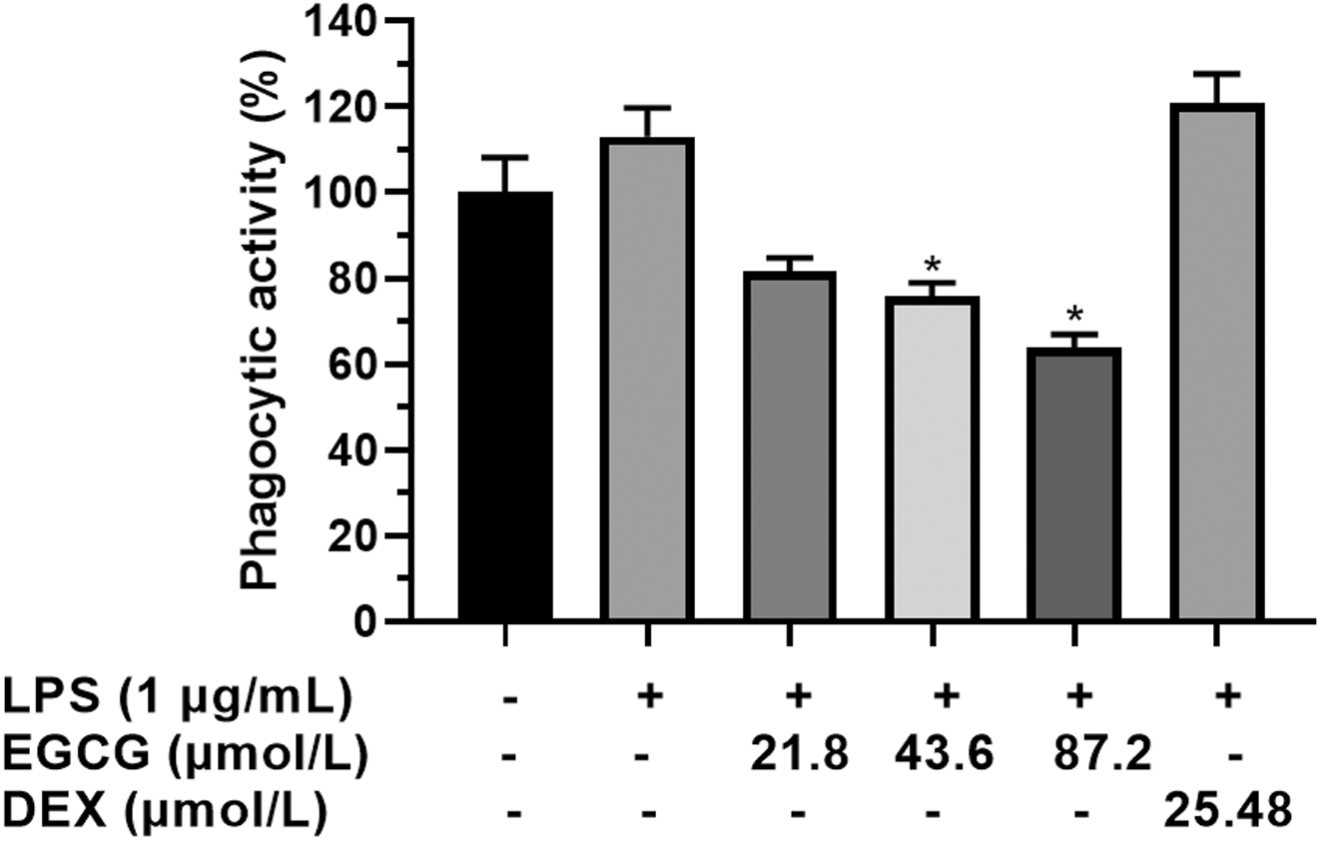
RAW264.7 macrophage cells (1 × 10^5^ cells) cultured and treated with EGCG and EGCG+LPS and DEX for 24 h. The effects of EGCG on LPS‐induced phagocytosis of RAW264.7 cells. *p < .05 versus LPS group. Each data point represents the mean ± SD (*n* = 3).

Although the LPS group could promote the phagocytic capacity of cells, there was no significant difference from the control group (Figure 4). Compared with the LPS group, DEX stimulated the phagocytic capacity of the cells, which were approximately 120% and EGCG significantly inhibited the phagocytic capacity of the cells at 21.8 μmol/L, 43.6 μmol/L, and 87.2 μmol/L. The phagocytic capacity was 95%, 89%, and 85% (*p* < .05), respectively, and the inhibitory effect of EGCG was significantly higher than the DEX group (Figure 4).

### 3.4 Effect of EGCG on LPS‐induced NO

**FIGURE 5:**
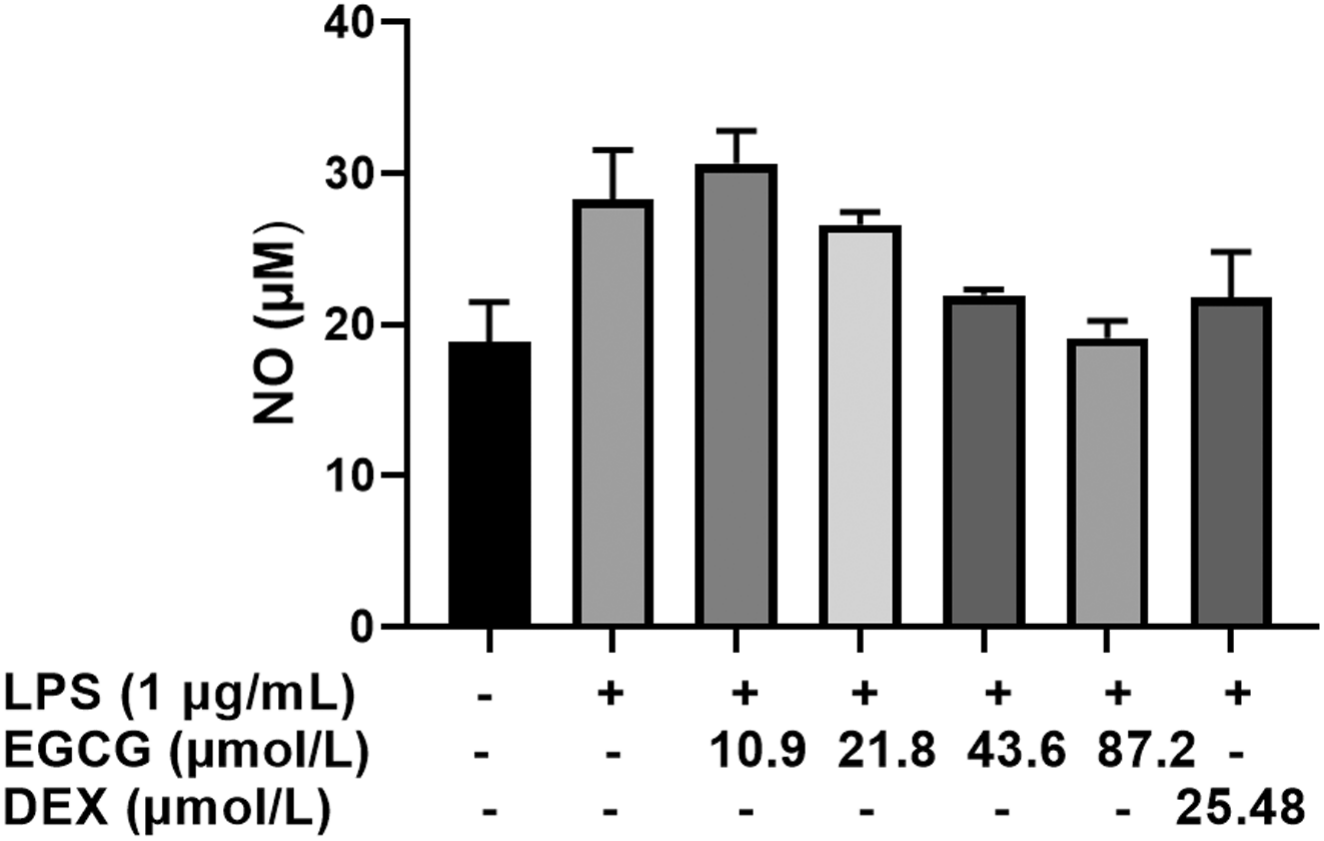
RAW264.7 macrophage cells (1 × 10^5^ cells) cultured and treated with EGCG and EGCG+LPS and DEX for 24 h. The effect of EGCG on LPS‐induced NO expression in RAW264.7 cells. Each data point represents the mean ± SD (*n* = 3).

After incubating RAW264.7 cells with LPS or LPS + EGCG for 24 h, the nitrite concentration in the culture solution was measured by the Griess method to characterize the content of NO. Compared with the control group, LPS can significantly increase the NO content by 55.55%. EGCG has a dose‐dependent effect on LPS‐induced NO production. EGCG at 10.9 μmol/L and 21.8 μmol/L failed to inhibit the production of NO, and there was no significant difference from the LPS group. 43.6 μmol/L and 87.2 μmol/L EGCG inhibited the production of NO induced by LPS, the content was almost reduced to the control group, which was significantly lower than that of the LPS group, and there was no significant difference from the control group. At the same time, the positive control (DEX) also decrease the NO content by about 21 μM, which is 25% lower than the LPS group (Figure 5).

### 3.5 EGCG inhibits ROS production in RAW264.7 macrophages

**FIGURE 6:**
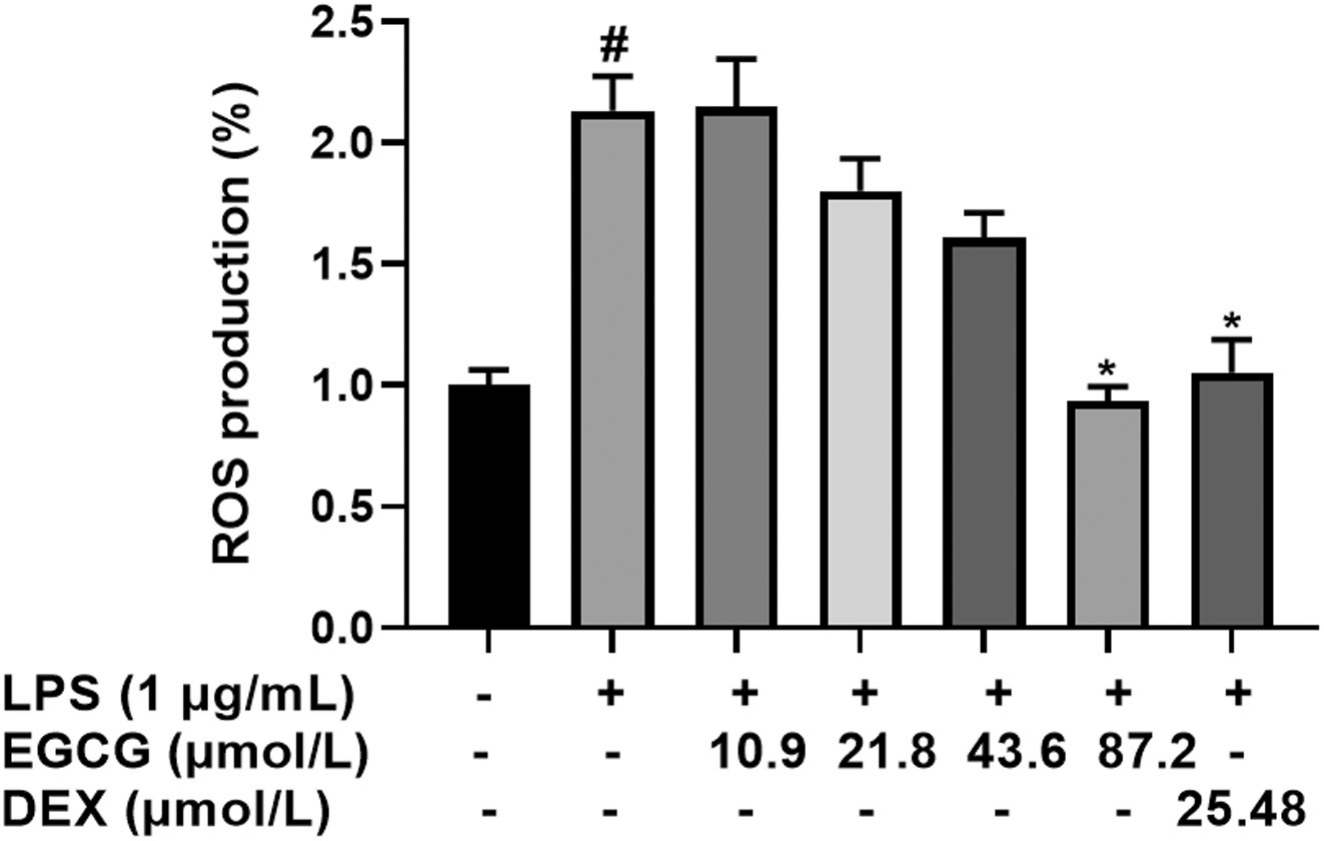
RAW264.7 macrophage cells (1 × 10^5^ cells) cultured and treated with EGCG and EGCG+LPS and DEX for 24 h. The effect of EGCG on LPS‐induced ROS expression in RAW264.7 cells. ^#^ *p* < .05 versus control group; *p < .05 versus LPS group. Each data point represents the mean ± SD (*n* = 3).

As can be seen from Figure 6, EGCG can inhibit the increase of ROS induced by LPS, and there is a certain dose–effect relationship. The content of ROS in the LPS group increased twice that of the control group, which significantly increased the expression of ROS (*p* < .05). DEX significantly inhibited the production of ROS induced by LPS with a content of about 100%, which was not significant. The relative ROS levels of 10.9 μmol/L and 21.8 μmol/L in the EGCG group were about 200% and 170%, respectively, and there was no significant difference from the LPS group. The relative ROS levels of the 43.6 μmol/L and 87.2 μmol/L EGCG groups were about 150% and 100%, respectively, which were significantly lower than those of the LPS group (*p* < .05).

### 3.6 Transcriptome analysis

**FIGURE 7:**
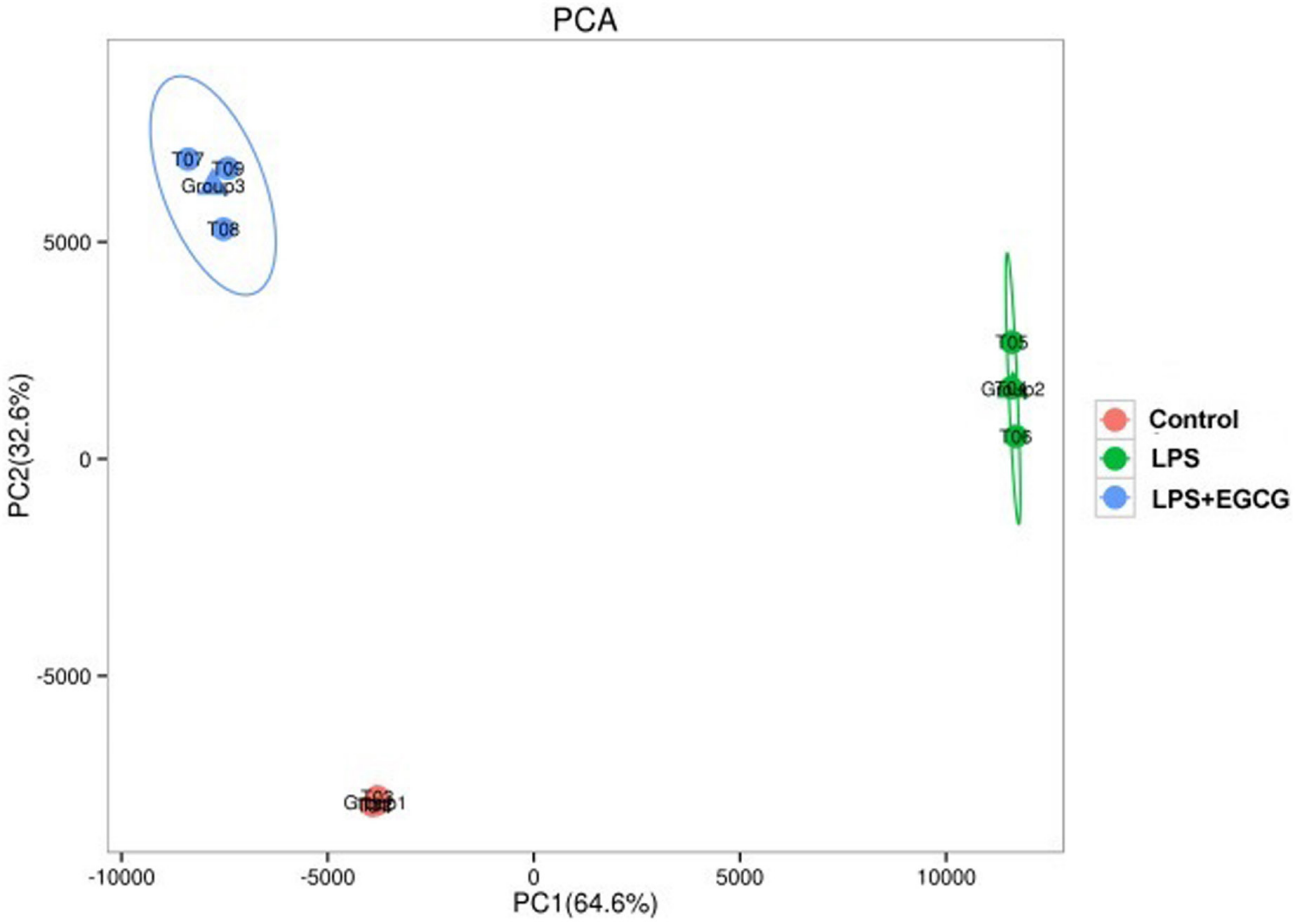
Principal component analysis of differential genes of control, LPS, and LPS + EGCG group.

A total of 23,939 genes were detected in this experiment, including 7346 differential genes. Principal component analysis (PCA) of differential genes found that the three groups are distributed in different areas, and each group does not interfere with each other, which can clearly distinguish the control group, LPS group, and LPS + EGCG group (Figure 7).

**FIGURE 8:**
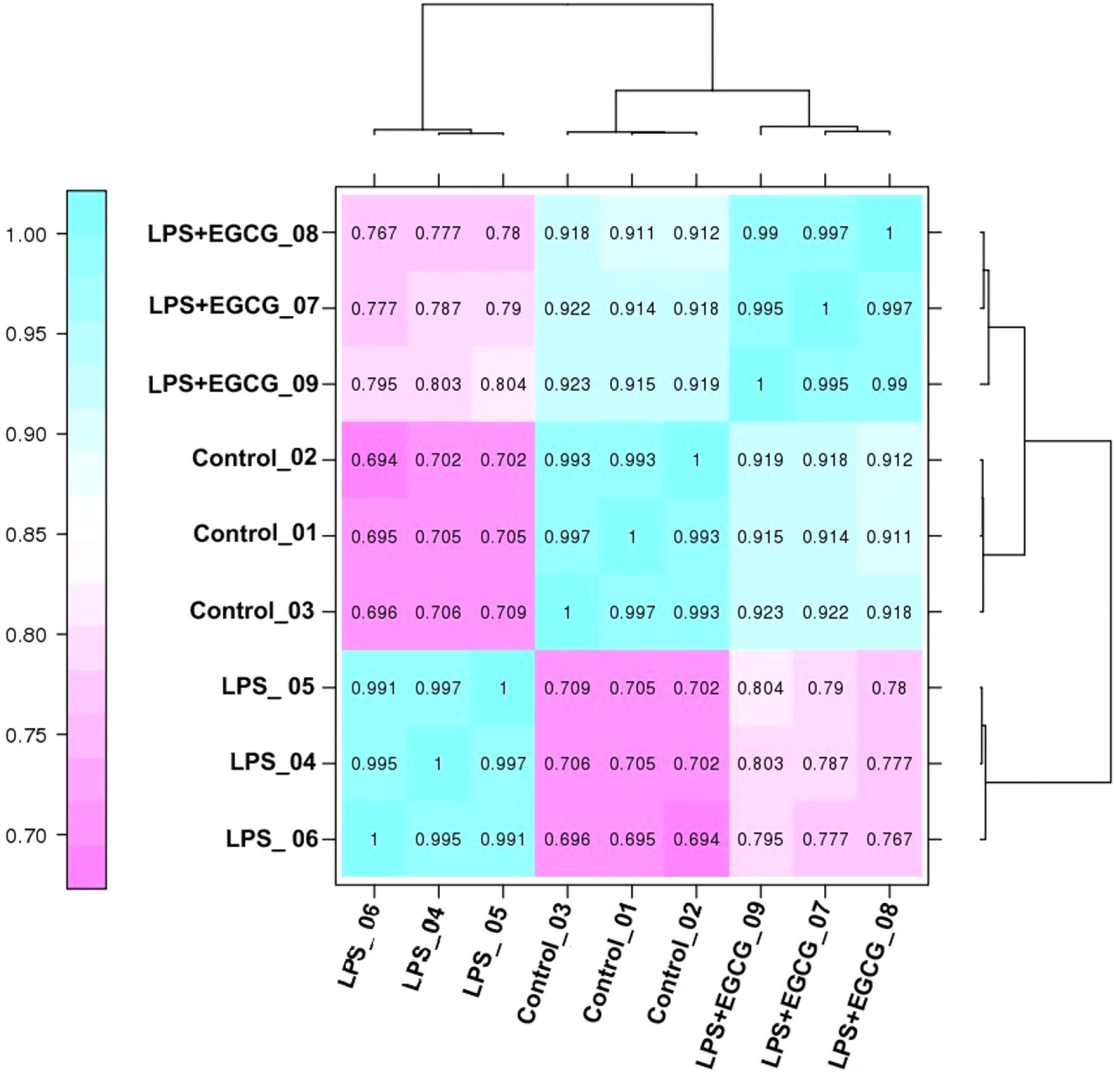
Correlation analysis of differential genes of control, LPS, and LPS + EGCG group.

At the same time, the correlation analysis revealed that the correlation between the control group and the LPS group was only about 0.7, the correlation between the LPS group and the LPS + EGCG group was about 0.78, and the correlation between the control group and the LPS + EGCG group was as high as about 0.91. This indicates that after EGCG treatment, the overall gene expression trend of cells tends to be that of normal cells (Figure 8).

**FIGURE 9:**
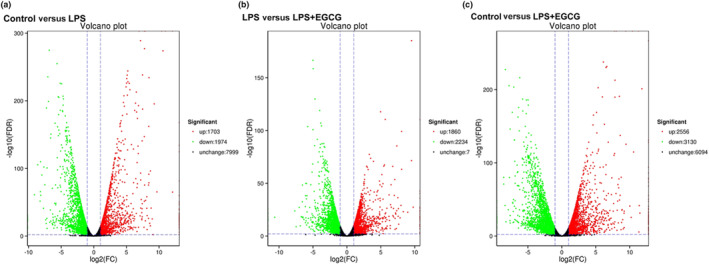
Volcano plot. Distinct transcriptome profile obtained after treating RAW 264.7 macrophages with EGCG, determined by RNA‐seq. Horizontal coordinates represent variations in mRNA expression levels. Longitudinal coordinates represent significant changes in mRNA expression levels. Red and green dots in the Volcano plot indicate mRNAs with increased and decreased expression levels.

In the differential gene expression volcano diagram, there are 3677 differential genes between the control group and the LPS group, including 1703 upregulated genes (red dots) and 1974 downregulated genes (green dots); there are 4094 differential genes in the LPS group and the EGCG + LPS group. Among them, there are 1860 upregulated genes and 2234 downregulated genes. The control group and the LPS + EGCG group have a total of 5686 differential genes, 2556 upregulated genes, and 3130 downregulated genes (Figure 9).

**FIGURE 10:**
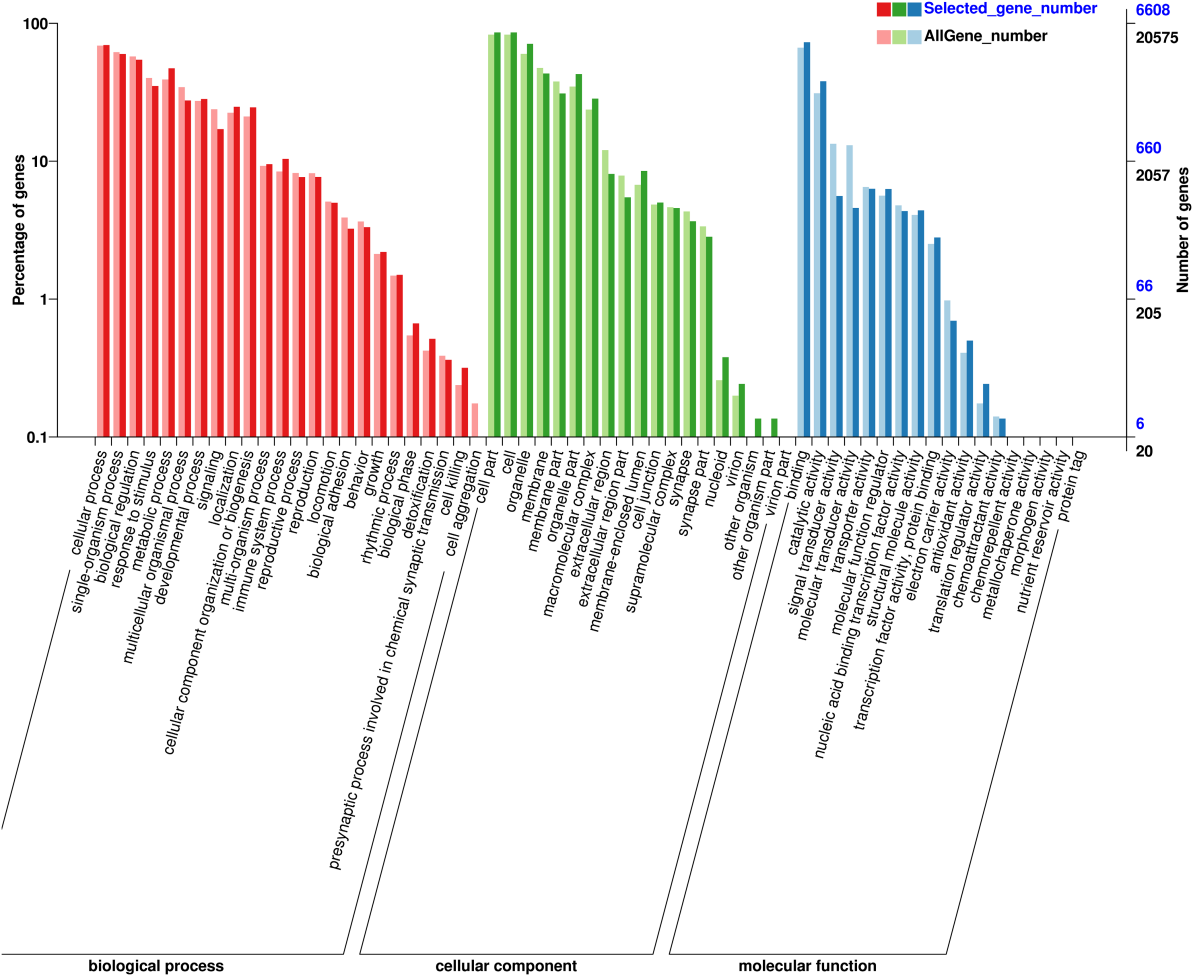
Gene ontology (GO) classifications of DEGs across three comparisons. The Y‐axis represents the number of DEGs in a category. The results are divided into three main categories: biological process (BP), cellular component (CC), and molecular function (MF).

To study the effect of EGCG on the biological process (BP), cellular component (CC), and molecular function (MF) of RAW264.7 cells, the GO secondary function was obtained using the GO database Gene enrichment. Figure 9 shows that 24 BP, 18 MF, and 19 CC‐related processes are involved. In BP, we found that differential genes differ from all genes in terms of metabolic processes, multicellular biological processes, signals, immune system processes, biological stages, detoxification, and cell killing. In terms of CC, there are differences in gene enrichment in nucleoids, viruses, organelle parts, macromolecular complexes, extracellular regions, and luminal parts enclosed by membranes. In MF, the difference in gene transduction, molecular transformation, translation regulation, antioxidant activity, catalytic activity, and other genes are significantly different from those of all genes (Figure 10).

**FIGURE 11:**
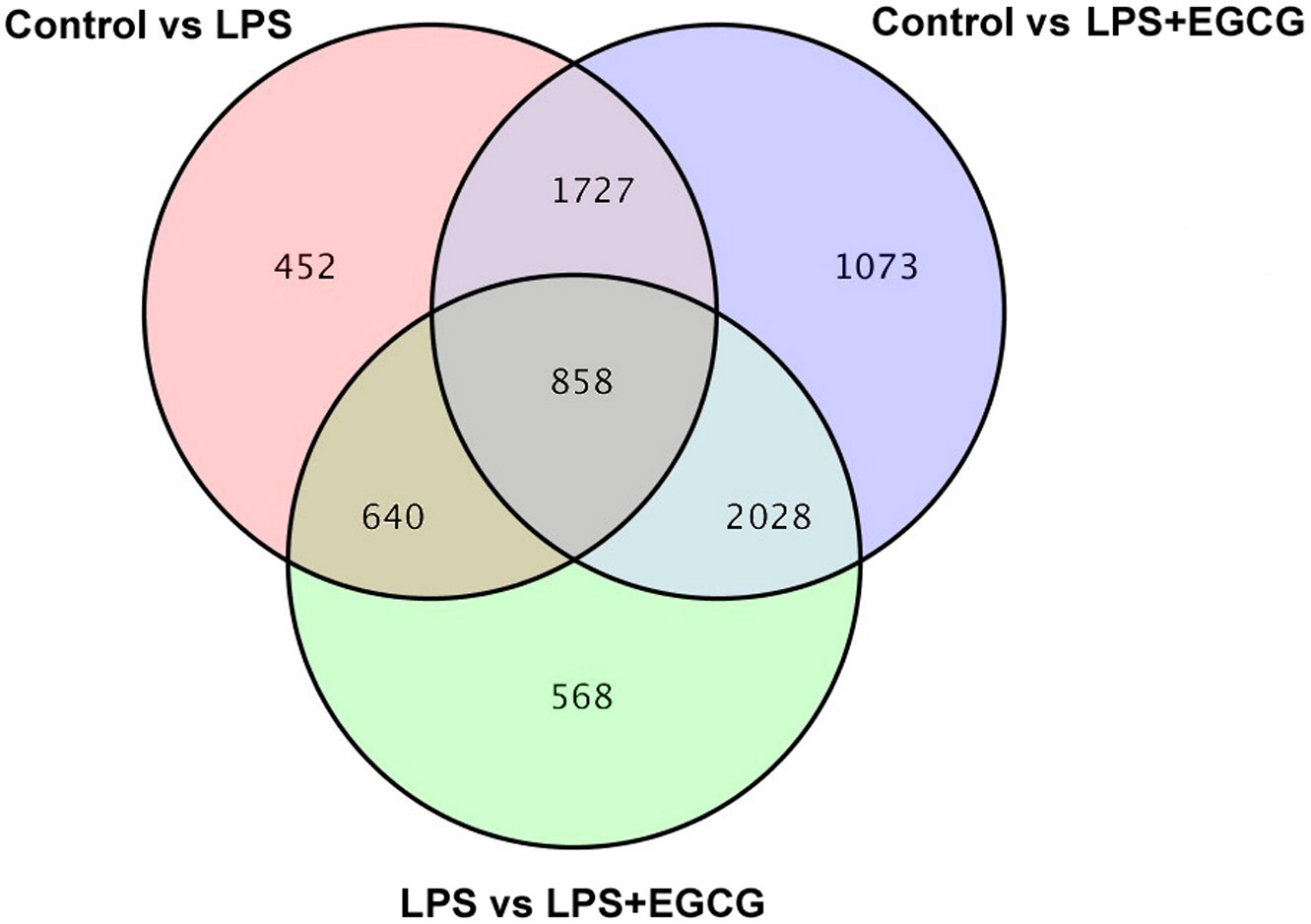
Venn diagram of differential genes. Venn diagram representing total number of genes identified among the groups.

Analysis of differentially expressed genes (DEGs) revealed that the control group and LPS group had 452 differential genes, the control group and LPS + EGCG group had 1073 differential genes, and the LPS group and LPS + EGCG group had 568 differential genes. The three groups involved 858 common differential genes, indicating that LPS induces changes in these genes, and EGCG will also regulate gene expression changes caused by LPS. After that, we further analyzed the differential genes shared by the three groups, which laid the foundation for studying the possible ways of EGCG (Figure 11).

**FIGURE 12:**
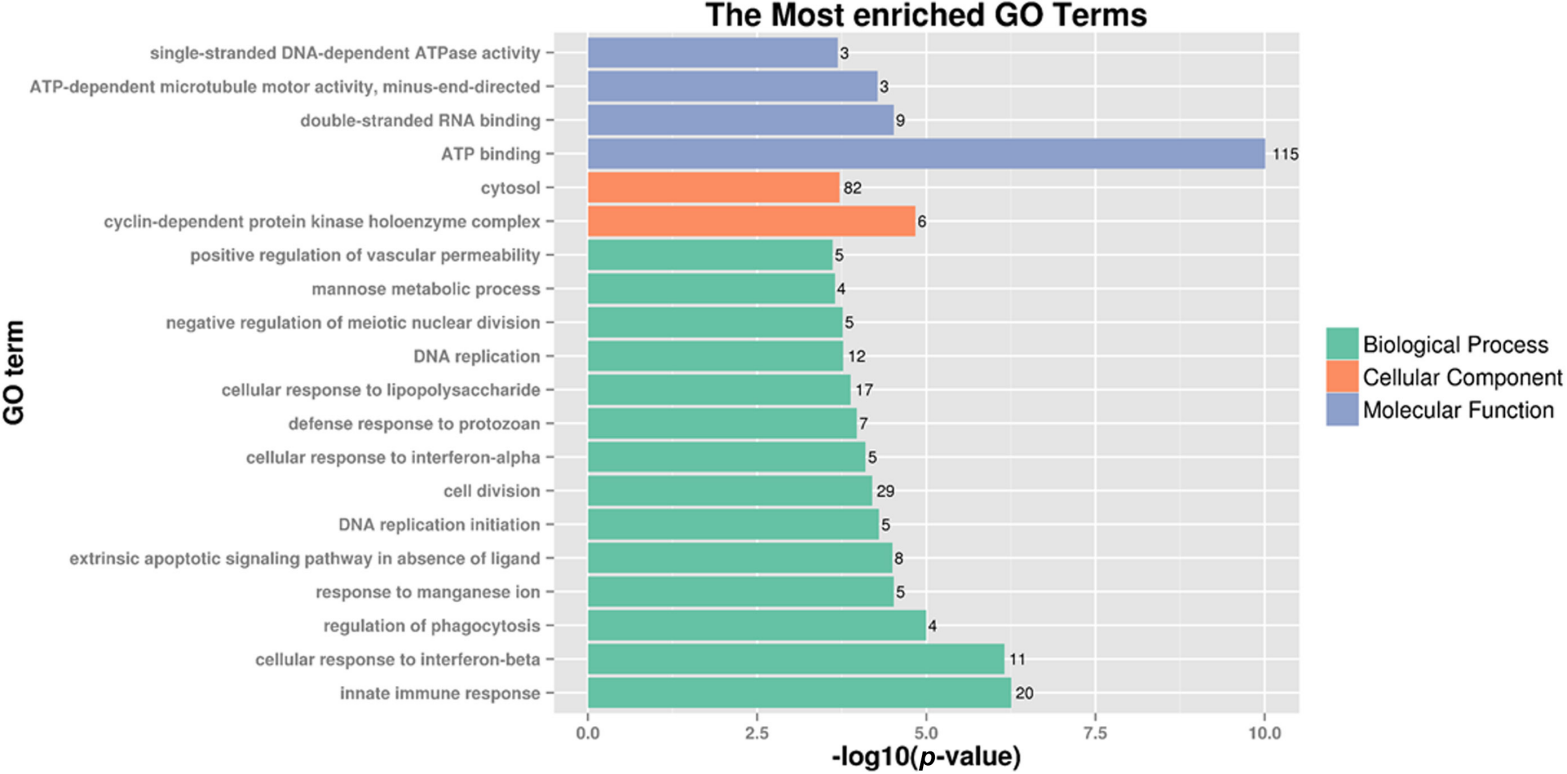
Analysis of GO pathway enrichment of differential genes. Gene ontology (GO) analysis of significant genes. Bar plots displaying enriched biological processes, cellular components, and molecular function. The plots show significantly enriched GO terms.

GO enrichment analysis showed that the three groups of differential genes were enriched in the GO function annotations for the 20 most significant related functions. Among them, 115 differential genes are involved in the Adenosine triphosphate (ATP) binding process. Secondly, the innate immune response involves 20 differential genes, the cell response to LPS involves 17 differential genes, the cell response to interferon‐β involves 11 differential genes, and the cell response to interferon‐α involves five differential genes, the regulation of phagocytosis involves four differential genes. These processes are closely associated with the inflammatory immune response of macrophage cells (Figure 12).

**FIGURE 13:**
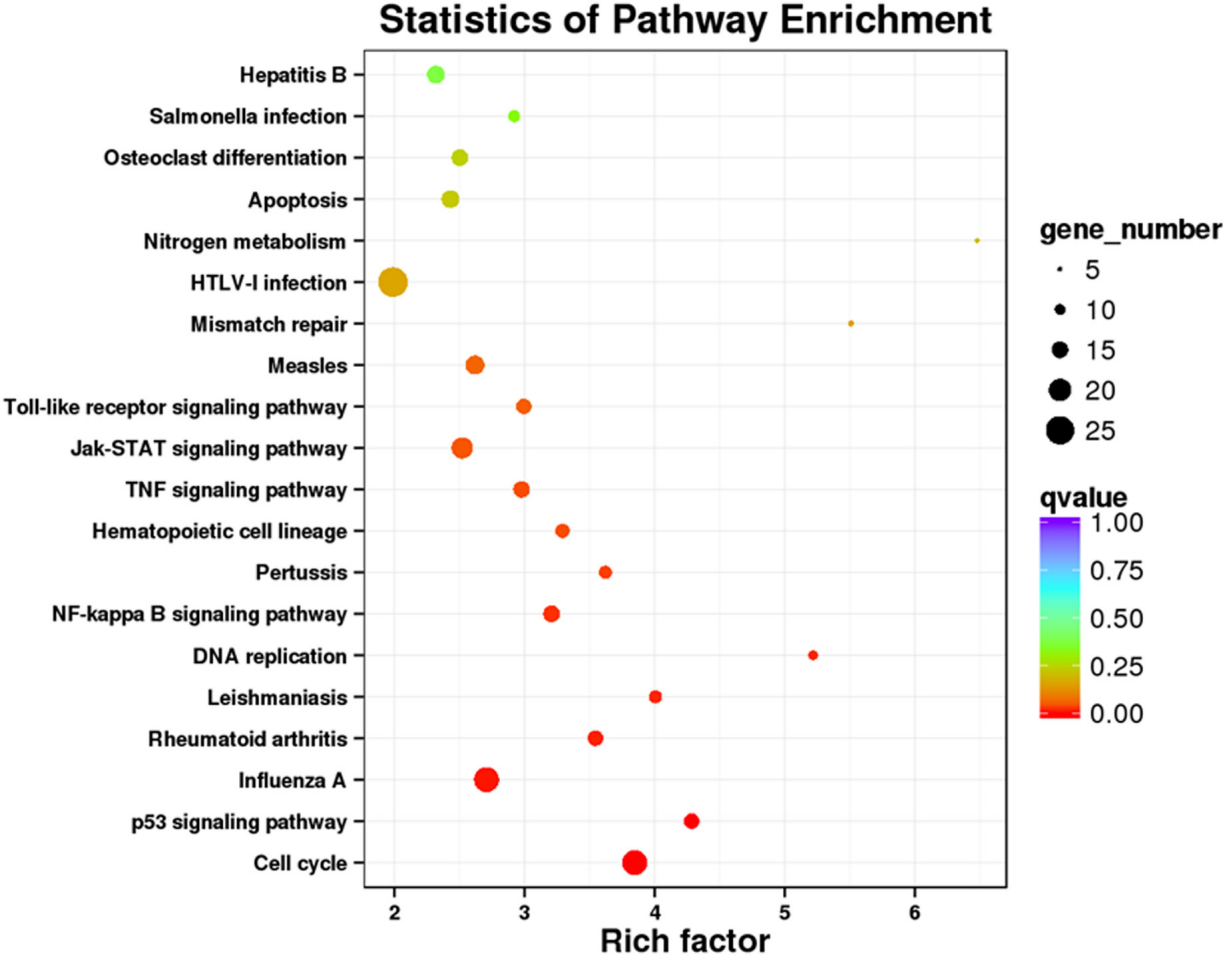
Analysis of KEGG pathway enrichment of differential genes. The color depth of nodes refers to the *p*‐value. The size of nodes refers to the number of genes.

We conducted KEGG enrichment analysis on the three groups of differential genes, and then evaluated the enrichment degree of KEGG by enrichment factor (rich factor), q‐value, and gene quantity and displayed the top 20 most enriched signal pathways. The greater the enrichment factor, the more significant the enrichment level of differential genes in this pathway. Among them, the classic inflammation pathway NF‐κB is the most significant among the inflammation‐related pathways, and its enrichment degree is about 3.1. Secondly, there are the TNF‐alpha signaling pathway, Toll‐like receptor signaling pathway, etc. (Figure 13).

**FIGURE 14:**
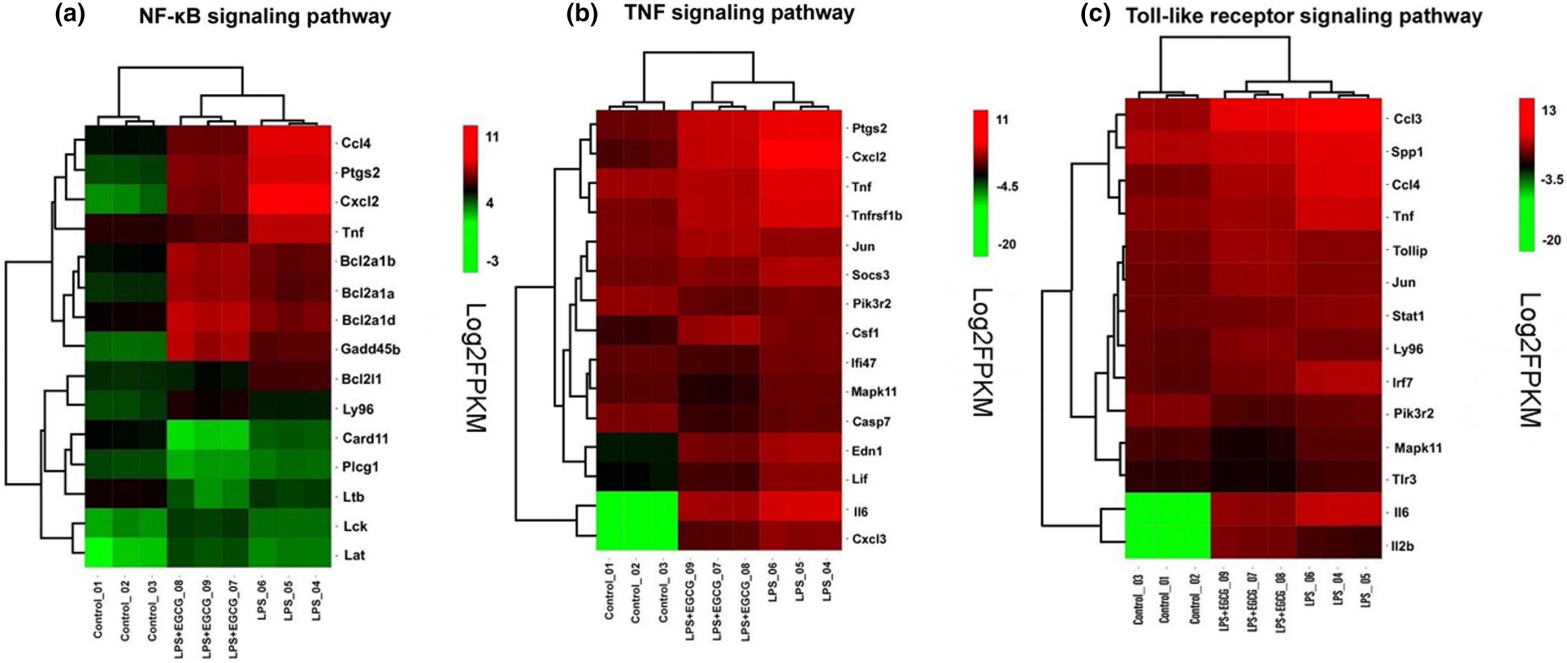
Heatmap analysis of differential genes involved in NF‐κB, TNF, and Toll‐like receptor signaling pathways.

Heatmap analysis of the NF‐κB pathway shows that EGCG inhibits LPS‐induced upregulation of Ccl4, Cxcl2, Ptgs2, TNF, Bcl21, Card11, Ltb, and Plcg1. Among them, Ptgs2 is a promoter of tumor and cancer formation, and Cxcl2 is a chemokine, which is closely related to inflammation. CD40 can activate PI3K, Rel/NF‐κB transcription factors, induce proteins such as Bcl‐xL and Cdk4, and inhibit Pidd1. p53 induces death domain proteins, causing inflammation, etc. In the TNF signaling pathway, LPS induces upregulation of Ptgs2, Cxcl2, TNF, Tnfsf13b, Socs3, lfi47, Mapk11, Edn1, Lif, IL‐6, and Cxcl3, and the expression of these genes is suppressed after EGCG intervention. In the Toll receptor signaling pathway, EGCG inhibits LPS‐induced upregulation of Ccl3, Spp1, Ccl4, TNF, STAT1, Irf7, Trl3, Mapk11, IIl12b, and IL‐6, thereby regulating the Toll‐like receptor signaling pathway to suppress inflammation. Tollip is a negative regulator of TLR4. EGCG can significantly increase its expression and inhibit the overexpression of TLR4 (Figure 14).

### 3.7 EGCG inhibited LPS‐induced cytokine expression

**FIGURE 15:**
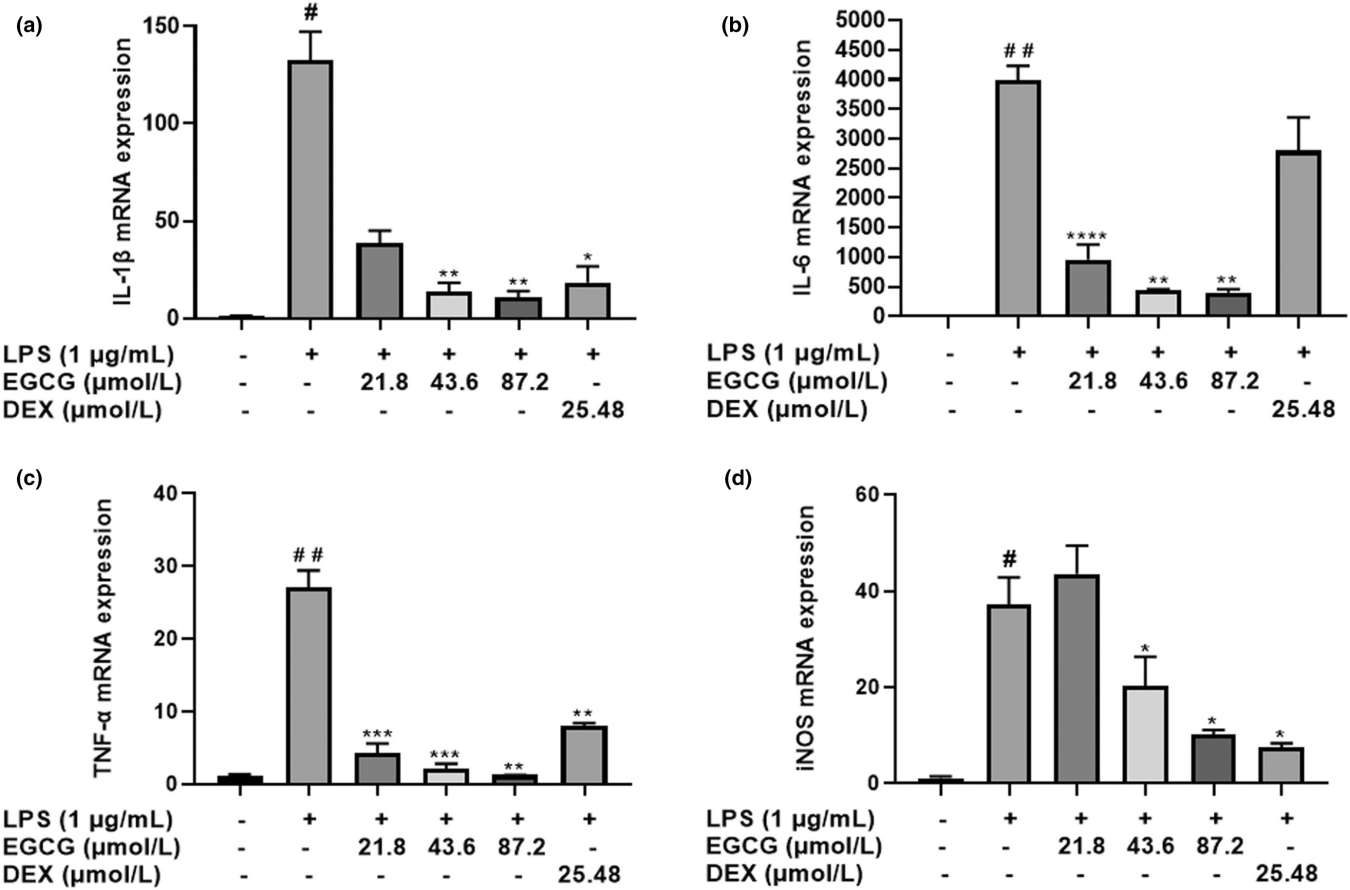
EGCG reduces cytokine expression. (a). IL‐1β, (b). IL‐6, (c). TNF‐α, and (d). iNOS. Data are represented as the mean ± SD (*n* = 3). # *p* < .01 versus control group, ^##^ *p* < .01 versus control group, *p < .05 versus LPS group, **p < .01 versus LPS group, ***p < .001 versus LPS group.

Raw 264.7 macrophage cells were stimulated with LPS and treated with various concentrations of EGCG; after which IL‐1β, IL‐6, TNF‐α, and iNOS expression were measured using RT‐PCR. Compared with the control group, LPS‐induced RAW264.7 cells exhibited IL‐1β mRNA expression over 100 times in 24 h. EGCG and DEX at various concentrations significantly inhibited IL‐1β mRNA expression (*p* < .01), the expression level is below 50 times (Figure 15a). The expression of IL‐6 in the LPS group was as high as 16,000 times, and EGCG at various concentrations significantly inhibits the expression of IL‐6. However, DEX treatment is not effective in inhibiting IL‐6 expression (Figure 15b). LPS treatment increased the TNF‐α level by 2700 times compared to the control group. The inhibitory effect of EGCG at various concentrations on LPS‐induced TNF‐α mRNA expression was very significant, it almost normalized the level (*p* < .001) (Figure 15c). iNOS level increased by 4000% in the LPS treatment group compared to normal control. 21.8 μmol/L EGCG rather increased iNOS level instead of lowering, 43.6 μmol/L, and 87.2 μmol/L EGCG and DEX significantly inhibited iNOS mRNA expression, which is significantly different from the LPS group (*p* < 0.05) (Figure 15d).

**FIGURE 16:**
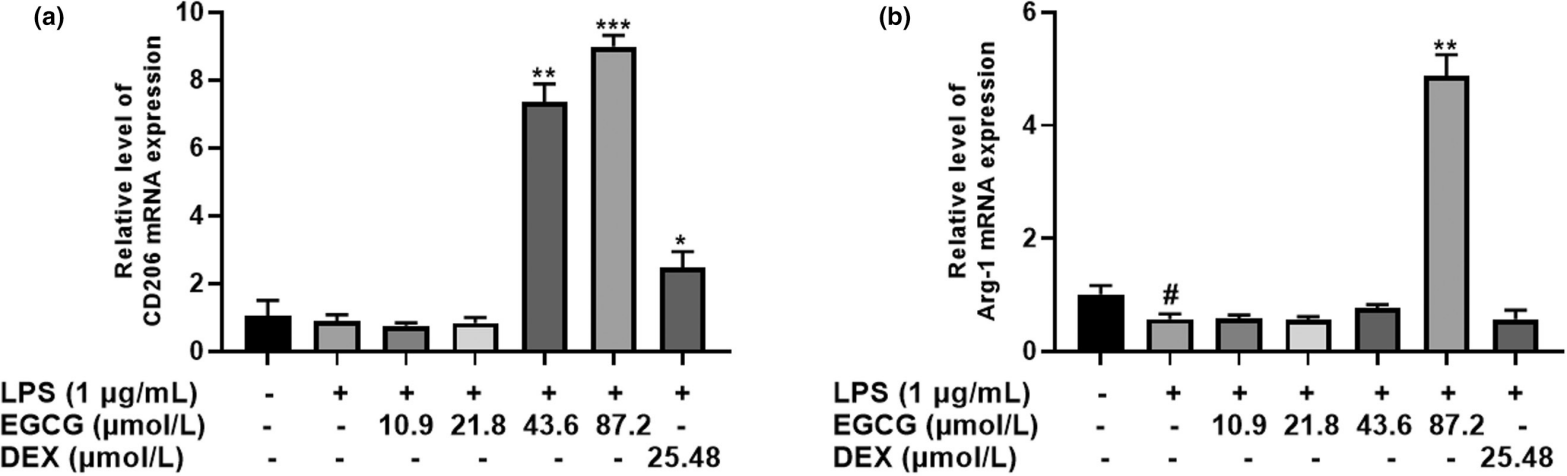
EGCG alters mRNA expression of cell surface markers. (a). CD206, and (b). Arg‐1. Data are represented as the mean ± SD (*n* = 3). ^#^ *p* < .01 versus control group, *p < .05 versus LPS group, **p < .01 versus LPS group, ***p < .001 versus LPS group.

By measuring the mRNA expression of Arg‐1 and CD206 (these are key markers of M2 macrophages) to study whether EGCG can regulate the transformation of RAW264.7 cells to M2 type. Under LPS stimulation, 43.6 μmol/L EGCG elevate CD206 mRNA expression by seven folds and 87.2 μmol/L by nine folds. Positive control, DEX (25.48 μmol/L) significantly increase CD206 expression levels by two folds. For Arg‐1, 43.6 μmol/L EGCG has no significant effect after LPS stimulation but 87.2 μmol/L EGCG significantly increased the mRNA expression of Arg‐1 by about five times (*p* < .05) (Figure 16).

### 3.8 Effect of EGCG on LPS‐induced NF‐κB signaling pathway

**FIGURE 17:**
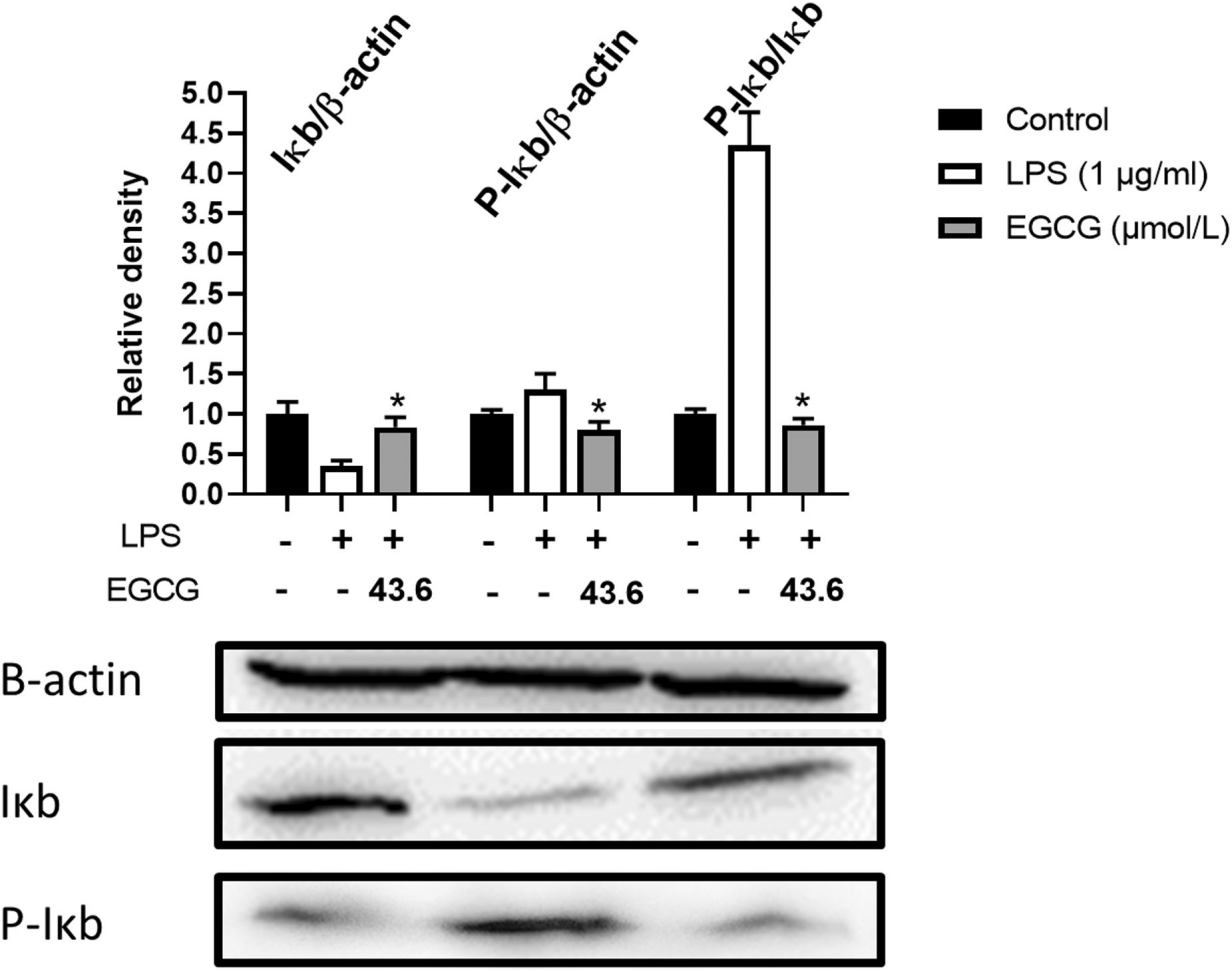
EGCG significantly inhibits LPS‐induced activation of IκB. Data are represented as the mean ± SD (*n* = 3). *p < .05 versus LPS group. The beta Actin band in this figure is the same band in Figure 20a because we have separated them in the same membrane.

The expression of IκB in the LPS group was significantly lower than that in the EGCG+LPS group, while the expression of activated P‐IκB was significantly higher than that in the EGCG+LPS group (*P* < .05). At the same time, the ratio of P‐IκB/IκB in the EGCG+LPS group was 75% lower than that of the LPS group, indicating that EGCG could significantly inhibit LPS‐induced activation of IκB, thereby inhibiting the activation of NF‐κB (*p* < .05) (Figure 17).

**FIGURE 18:**
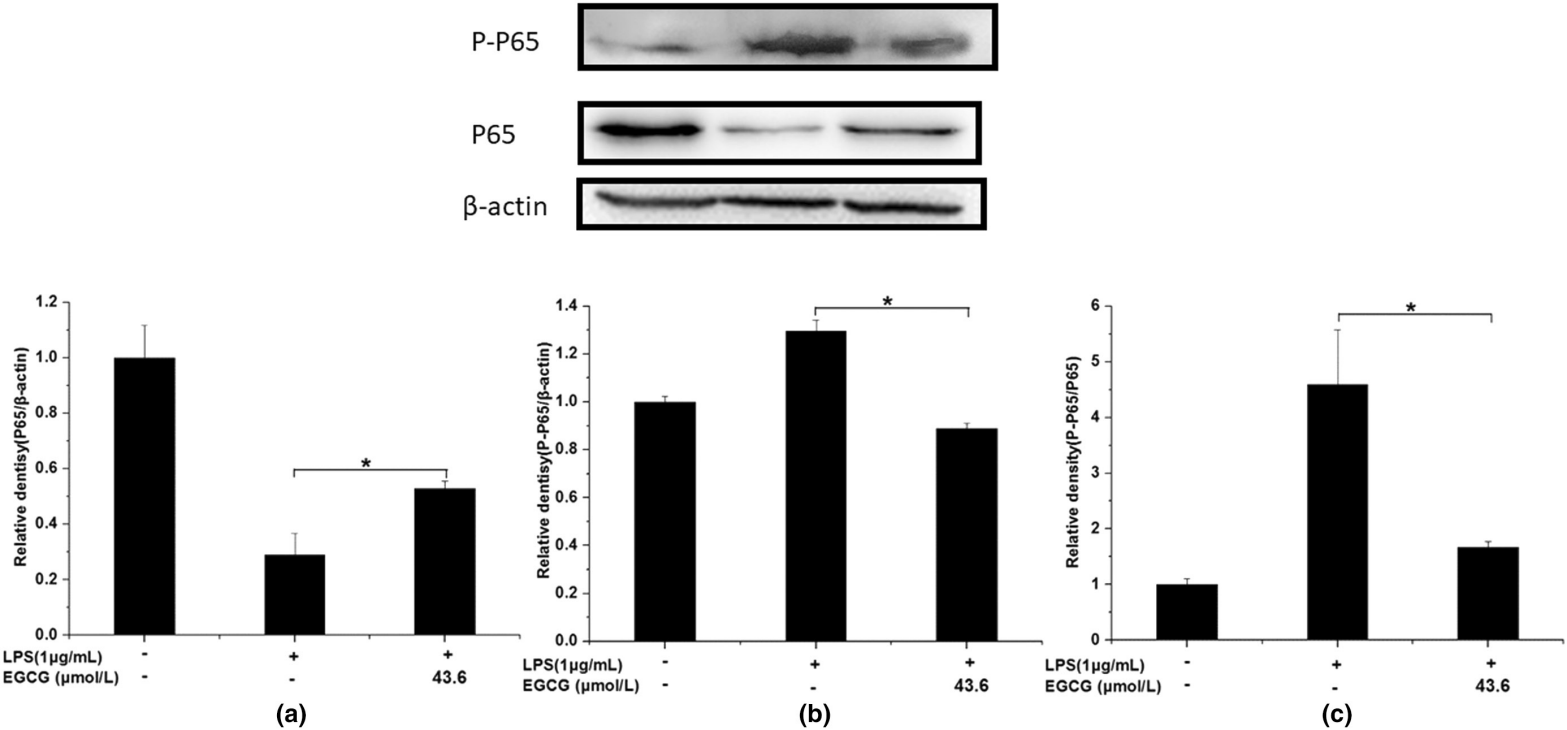
EGCG significantly inhibits the activation of P65 induced by LPS. Data are represented as the mean ± SD (*n* = 3). *p < .05 versus LPS group. The beta Actin band in this figure is the same band in Figure 19b because we have separated them in the same membrane.

**FIGURE 19:**
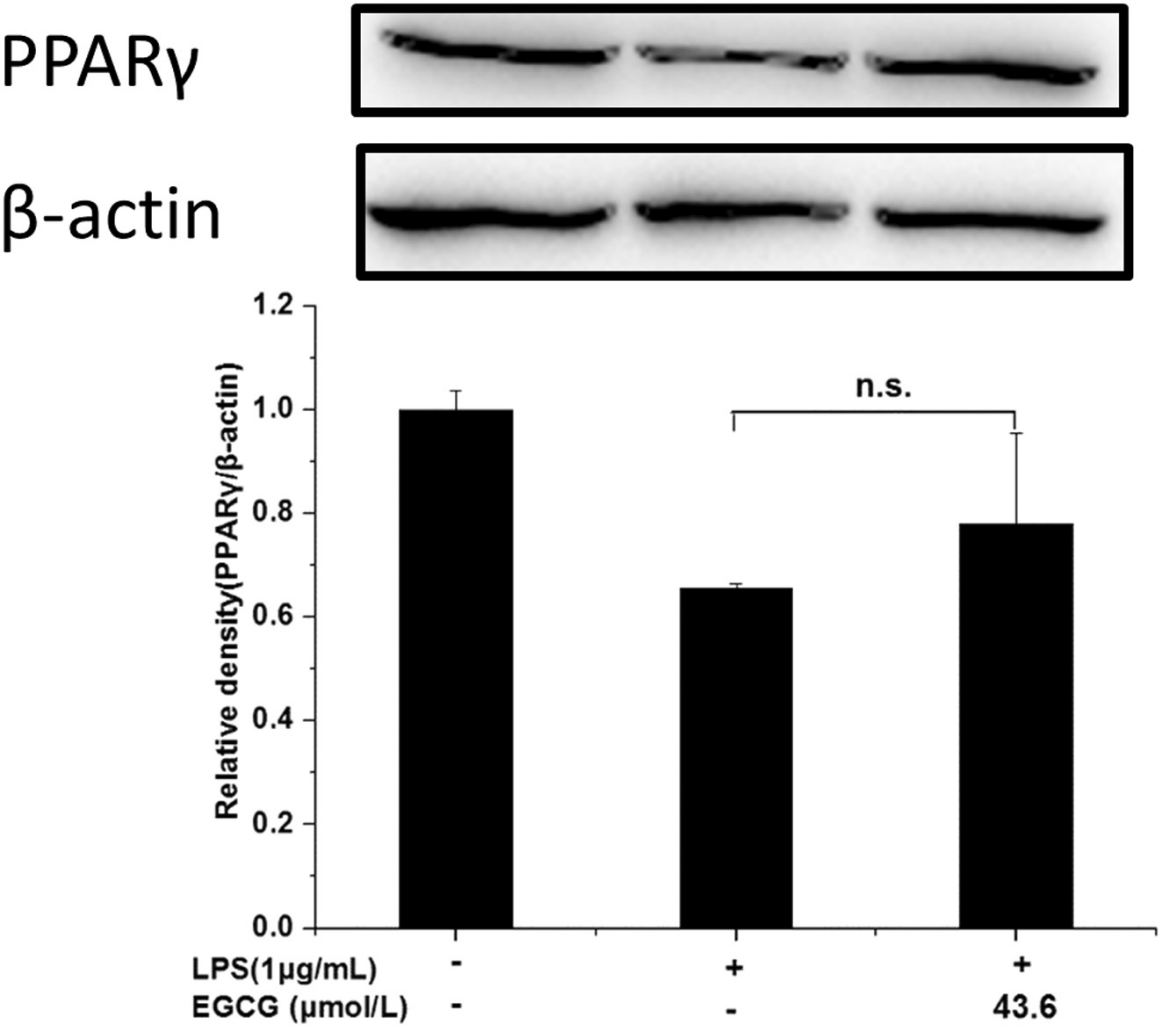
Effects of EGCG on PPARγ. Data are represented as the mean ± SD (n = 3).

Similarly, the expression level of P65 in the EGCG+LPS group was about twice that of the LPS group, while the expression level of P‐P65 was only about 2/3 of that in the LPS group. The expression of P‐P65/P65 in the EGCG+LPS group was significantly lower than that in the LPS group, about 1/2 times that of the LPS group, indicating that EGCG can significantly inhibit the activation of P65 induced by LPS, thereby exerting an inflammatory protective effect (*p* < .05) (Figure 18). However, EGCG could not significantly increase the expression of PPARγ (Figure 19).

**FIGURE 20:**
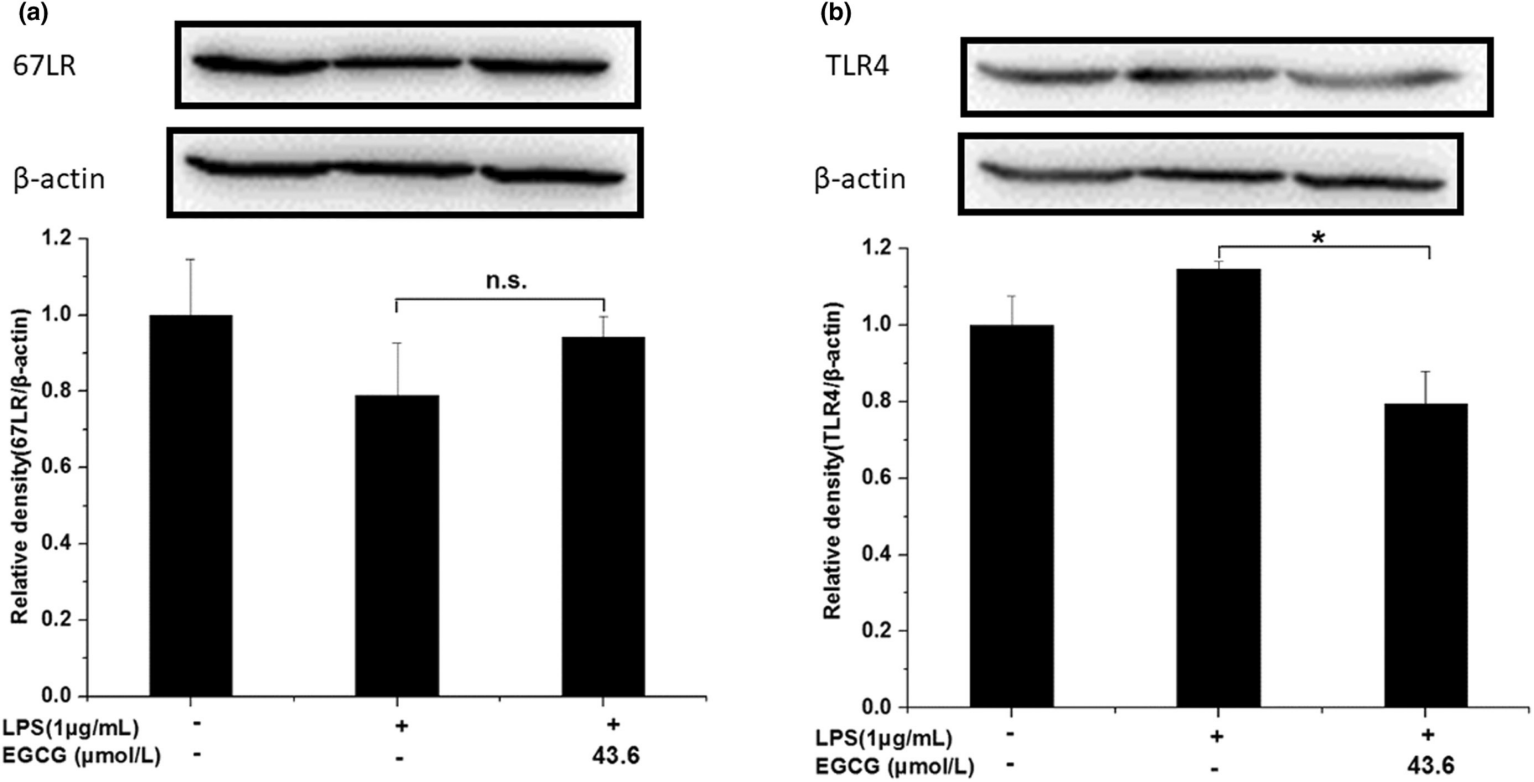
Effects of EGCG on 67LR (a) and TLR4 (b). Data are represented as the mean ± SD (*n* = 3). *p < .05 versus LPS group. The beta Actin band in this figure is the same band in Figure 18. The beta Actin band in this figure is the same band in Figure 19 because we have separated them in the same membrane.

Compared with the control group, LPS did not significantly affect the protein expression of 67LR. Similarly, there was no significant difference between the LPS group and the LPS + EGCG group as well (Figure 20a). LPS treatment elevated TLR4 expression, but it was also not statistically significant. Compared with the LPS group, EGCG downregulated the expression of TLR4 significantly (*p* < .05), and the expression level is about 70% of the LPS group, indicating that EGCG can exert an inflammatory protective effect by inhibiting the expression of TLR4 (Figure 20b).

## 4 DISCUSSION AND CONCLUSION

EGCG is the major part of green tea polyphenol that suppresses inflammation, oxidation, tumor, and apoptosis. In the present study, we demonstrated that EGCG, a bioactive polyphenol in green tea, suppressed the expression of LPS‐induced inflammatory cytokines in Raw 264.7 macrophage cells by mediating TLR4 and NF‐κB signaling pathways.

Neutrophils are the key player in the body's defense to combat inflammation. These neutrophils extrude pseudopods while performing their innate task. Pseudopods have many forms and their size and shape depend on the degree of polymerization of actin filaments (Rocheleau et al., 2016). EGCG significantly inhibited the formation of cell pseudopods, and the effect was better than DEX. Cui et al. (2019) also observed similar morphological changes in macrophages. The cells in the control group aggregated and showed a round shape. After LPS treatment, the cell adhesion increased and the body shape increased, forming a long and slender pseudopod protrusion.

Macrophages participate actively in the immune response to fight foreign stimuli, pathogens, and damaged cells by engulfing them. In this experiment, EGCG can significantly reduce the phagocytic capacity of RAW264.7 cells after LPS stimulation. This may be because, after EGCG intervention, it significantly reduced inflammatory factors such as IL‐1β and IL‐6, reducing the damage of the inflammatory response to the body by the ability of macrophages to engulf harmful stimuli also decreases. When *Bougainvillea xbuttiana* extract inhibited LPS‐induced macrophage phenotypic transformation, it also showed that *Bougainvillea xbuttiana* extract had a similar effect on the phagocytic capacity of RAW264.7 cells (Arteaga Figueroa et al., 2017).

At the same time, EGCG can significantly inhibit the abnormal increase of ROS induced by LPS. In our experiment, the inhibitory effect of EGCG at various concentrations on NO content was consistent with iNOS mRNA expression. This experiment also confirmed that it can reduce body inflammation and reduce the expression of most inflammatory factors. However, the results of this experiment show that DEX does not inhibit the expression of IL‐6 mRNA induced by LPS. It is undeniable that its anti‐inflammatory effect may be because DEX is not sensitive to IL‐6, mainly by inhibiting other inflammatory factors to produce an anti‐inflammatory effect. Similarly, a large number of studies have also used DEX as a positive control. Al‐Harbi et al. (2016) also found that DEX could not downregulate the increase of IL‐6 content in Balb/c mice induced by LPS. Muniandy et al. (2018) also found that DEX intervention increases the content of IL‐6 in RAW264.7 cells, which is about four times that of the LPS group which is similar to our findings (Muniandy et al., 2018).

We performed transcriptome sequencing analysis on three sets of cell samples (control, LPS, and LPS + EGCG), and 7346 differential genes were obtained. The three groups of differential genes were screened and analyzed based on GO function enrichment terms. The results show that the ATP binding pathway is significantly affected. In mitochondria, adenosine diphosphate (ADP) is consumed to produce ATP and oxygen consumption is blocked to produce O_2−_, and superoxide anions play a central role in ROS production (Piechota‐Polanczyk & Fichna, 2014). About 90% of ROS in cells are produced by mitochondrial oxidative phosphorylation and imbalance of these leads to the release of inflammatory factors such as TNF‐α and IL‐1β, triggering innate immunity, eventually causing immune responses (Gao et al., 2008; Yamauchi et al., 2008). The activation of the TLR‐mediated signaling pathway is also closely related to ROS generation. In LPS/TLR4‐mediated inflammation, inhibition of ROS production helps reduce LPS‐induced NF‐κB activation (Ryan et al., 2004). Therefore, a reasonable adjustment of the relationship between ROS and ATP, and the balance of ROS production can help suppress inflammation and maintain the health of the body.

KEGG enrichment analysis revealed that the NF‐κB pathway was significantly affected in the common differential genes. KEGG enrichment analysis found that NF‐κB, a classic inflammation‐related pathway, is significantly higher (involved total of 15 differential genes), and the TNF signaling pathway and TLR signaling pathway involved 15 and 14 differential genes, respectively. He et al. (2017) and Wang et al. (2014) reviewed the signal pathways involved in macrophage polarization, including STAT, NF‐κB, PPARγ, IRF, etc. (He et al., 2017; Wang et al., 2014), which is consistent with our sequencing results. At the same time, it also has a significant impact on the TNF signaling pathway and the TLR signaling pathway. TLRs are pattern recognition receptors distributed on the surface of B‐lymphocytes, macrophages, and other cells, and exclusively expressed in the natural immune system. The activation of TLRs can induce an immune response, which is beneficial to the body's resistance to pathogenic microorganisms, but excessive activation induces the overexpression of inflammatory factors and aggravates the development of immune diseases. At the same time, the combination of LPS and TLR4 causes the expression of TNF‐α, which also causes the activation of the TNF signaling pathway.

In this experiment, there was no significant difference between the 67LR protein expression of the LPS group and the LPS + EGCG group. At the same time, EGCG significantly inhibited LPS‐induced TLR4 expression (*p* < .05). Similar to our findings, Bao et al. (2015) showed that in LPS‐induced 3 T3‐L1 adipocytes, 67LR protein expression remains unaffected after EGCG treatment (Bao et al., 2015). Baek et al. (2019) found that EGCG can significantly downregulate the expression of TLR4 in human aortic endothelial cells (HAECs) induced by LPS (Baek et al., 2019). Byun et al. (2010) showed in macrophages that EGCG alone could downregulate the protein expression of TLR4 for 24 h, and EGCG did not inhibit the expression of TNF‐α and other inflammatory factors after incubating the cells with 67LR (Byun et al., 2010). This indicates that LPS does not stimulate the cells to cause inflammation by reducing the expression of 67LR but it may combine with TLR4 to produce more factors that are inflammatory and induce inflammation. At the same time, EGCG may play an anti‐inflammatory role by reducing the expression of TLR4, accelerating the degradation of TLR4, and affecting the downstream signal pathway of TLR4 (Kumazoe et al., 2017). NF‐κB can induce the transformation of macrophages to M1 type, produce a large number of inflammatory factors, and promote the body's inflammatory response. The experimental results show that EGCG can significantly inhibit LPS‐induced IκB activation, and the ratio of P‐IκB/IκB is 1/4 times that of the LPS group (*p* < .05); at the same time, EGCG can also significantly inhibit LPS‐induced P65 activation, the expression level of P‐P65/P65 was about 1/2 times that of the LPS group (*p* < .05). Li et al. (2017) also observed that EGCG can inhibit the expression of NF‐κB in macrophages, thereby inhibiting the expression of matrix metalloproteinase‐9 and monocyte chemoattractant protein‐1 (Li et al., 2017). Similarly, Joo et al. (2012) also reported that EGCG can inhibit the expression of nuclear protein NF‐κB P65 induced by LPS in bone‐marrow‐derived macrophages (BMMs) (Joo et al., 2012). This indicates that EGCG can significantly inhibit the activation of NF‐κB and thus suppress cell inflammation. PPARγ is closely associated with inflammation, and it can exert anti‐inflammatory effects by inhibiting inflammatory signaling pathways such as NF‐κB, activator protein‐1 (AP‐1), JAK–STAT, etc. (Bright et al., 2008; Dana et al., 2019). PPARγ can interact with P65 to prevent protein activation by inhibiting the activation of NF‐κB (Yin et al., 2014). In this experiment, the EGCG+LPS group could not significantly increase the expression of PPARγ. The difference is that Jin et al. (2020) found that fucoxanthinol can significantly upregulate the downregulated PPARγ expression in LPS‐induced macrophage cells, thereby, directly and indirectly, downregulating NF‐κB to suppress inflammation (Jin et al., 2020). This shows that there may be differences in the action pathways of EGCG and fucoxanthinol on macrophages. In macrophages, EGCG may not indirectly inhibit NF‐κB activation by upregulating PPARγ and regulating the conversion of macrophages between M1 and M2 types.

In conclusion, the present study demonstrated that EGCG administration protected against LPS‐induced inflammation in Raw 264.7 macrophage cells. EGCG inhibited LPS‐induced enhancement of cell phagocytosis and the upregulation of pro‐inflammatory factors. EGCG can significantly inhibit LPS‐induced upregulation of P‐IκB and P‐P65, and inhibit the activation of the NF‐κB pathway. Additionally, EGCG does not upregulate the expression of PPARγ, indicating that EGCG may not promote the transformation of macrophages to M2 type by increasing the expression of PPARγ. The action mechanism of EGCG is yet to be fully elucidated and needs more exploration in both in vivo and in vitro models. We conclude that EGCG may not promote the transformation of M1‐type macrophages to M2 type by increasing the expression of PPARγ. Combined with transcriptome data, further study is needed to grasp what substances and signal pathways EGCG regulates to play a role. At the same time, in vivo experiments are needed to further study the function and mechanism of EGCG.

## AUTHOR CONTRIBUTIONS

**Imam Hossen:** Conceptualization (equal); formal analysis (equal); investigation (equal); methodology (equal); validation (equal); writing – review and editing (equal). **Zhang Kaiqi:** Conceptualization (equal); data curation (equal); formal analysis (equal); investigation (equal); methodology (equal); software (equal); validation (equal). **Wu Hua:** Conceptualization (equal); funding acquisition (equal); investigation (equal); methodology (equal); resources (equal); software (equal); supervision (equal); visualization (equal); writing – review and editing (equal). **Junsong Xiao:** Conceptualization (equal); data curation (equal); formal analysis (equal); funding acquisition (equal); investigation (equal); methodology (equal); project administration (equal); resources (equal); supervision (equal); writing – original draft (equal); writing – review and editing (equal). **Mingquan Huang:** Methodology (equal); resources (equal); software (equal); validation (equal). **Yanping Cao:** Funding acquisition (equal); project administration (equal); resources (equal); supervision (equal).

## Acknowledgements

We express our gratitude towards Gao Zhipeng, Song Jingyi, Lv Wenwen and Ding Zhiqian for their assistance in the experimental processes including western blotting and transcriptome analysis.

## FUNDING INFORMATION

This research was supported by Beijing Natural Science Foundation (Grant No. 6212002) and Beijing municipal education commission general project (KM202010011010), and National Key Research and Development Program of China (Grant No. 2016YFD0400801).

## CONFLICT OF INTEREST STATEMENT

The authors declare no conflict of interest.

## Data Availability

The data that support the findings of this study are available on request from the corresponding author. The data are not publicly available due to privacy or ethical restrictions.
